# Synthesis of 3-aminocoumarin-*N*-benzylpyridinium conjugates with nanomolar inhibitory activity against acetylcholinesterase

**DOI:** 10.3762/bjoc.14.231

**Published:** 2018-10-02

**Authors:** Nisachon Khunnawutmanotham, Cherdchai Laongthipparos, Patchreenart Saparpakorn, Nitirat Chimnoi, Supanna Techasakul

**Affiliations:** 1 Laboratory of Organic Synthesis, Chulabhorn Research Institute, 54 Kamphaeng Phet 6, Talat Bang Khen, Lak Si, Bangkok 10210, Thailand; 2 Department of Industrial Chemistry, Faculty of Applied Science, King Mongkut's University of Technology North Bangkok, Bangkok 10800, Thailand; 3 Department of Chemistry, Faculty of Science, Kasetsart University, Bangkok 10900, Thailand,; 4 Laboratory of Natural Products, Chulabhorn Research Institute, 54 Kamphaeng Phet 6, Talat Bang Khen, Lak Si, Bangkok 10210, Thailand

**Keywords:** acetylcholinesterase inhibitor, 3-aminocoumarin, *N*-benzylpyridinium, dual binding site inhibitor, synthesis

## Abstract

A series of 3-amino-6,7-dimethoxycoumarins conjugated with the *N*-benzylpyridinium moiety through an amide-bond linkage was synthesized and evaluated for their acetylcholinesterase inhibitory activity. A number of the benzylpyridinium derivatives exhibited potent activities with inhibitory concentration (IC_50_) values in the nanomolar concentration range. Among them, the 2,3-difluorobenzylpyridinium-containing compound was the most potent inhibitor with an IC_50_ value of 1.53 ± 0.01 nM. Docking studies revealed that the synthesized compounds inhibit the target enzyme by a dual binding site mechanism whereby the coumarin portion binds with the peripheral anionic site while the *N*-benzylpyridinium residue binds with the catalytic anionic site of the enzyme.

## Introduction

An increasing number of countries are facing a rapid growth of the elderly population. The birth rates of many countries, such as China, Japan, and Thailand are lower than the number theoretically required for the replacement of successive generations, resulting in an aging population [1]. According to a United Nation report, the number of people over the age of 60 is expected to more than double by 2050, increasing from 962 million globally in 2017 to 2.1 billion in 2050, and exceed the number of younger people. As people age, they become susceptible to ageing-related health issues, such as arthritis, heart disease, cancer, diabetes and Alzheimer’s disease, all of which can potentially decrease the quality of life. Dementia, commonly found among the elderly, can be caused by the Alzheimer’s disease (AD) – a progressive neurodegenerative disorder. The Alzheimer’s Association reported that one in nine people over the age of 65 suffers from AD [2]. According to this report, approximately 46.8 million people worldwide lived with dementia in 2015, and this number is predicted to reach 75 million in 2030 and 131.5 million in 2050. AD is a chronic condition that remains incurable; most of the existing treatments only delay the onset or further advancement of AD.

**Figure 1:**
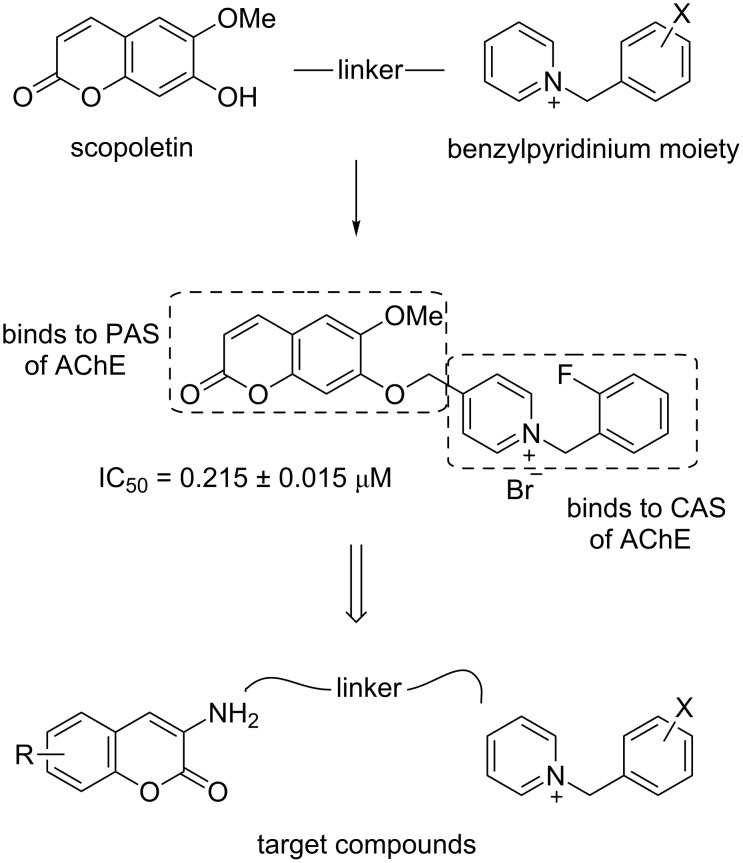
Design of the target compounds.

Among the current hypotheses for the treatment of AD, inhibition of acetylcholinesterase enzyme (AChE), which is responsible for the degradation of the neurotransmitter acetylcholine (ACh), is the most widely accepted hypothesis. AChE inhibition can increase ACh levels in the synaptic clefts and then alleviate the cognitive deficit in AD patients. The design of novel acetylcholinesterase inhibitors (AChEIs) has been mostly based on a dual-binding site strategy whereby the designed molecules simultaneously bind to amino acid residues present in both the catalytic anionic site (CAS) and the peripheral anionic site (PAS) of AChE. The CAS is located deeply inside the narrow gorge and is responsible for ACh hydrolysis. On the other hand the PAS is located around the entrance of the active site gorge and is responsible for transient substrate binding before reaching the active site. Especially PAS plays a crucial role in β-amyloid (Aβ) fibrillogenesis in AD patients by forming stable AChE–Aβ complexes [3–4]. Thus, these types of effects may render them potential therapeutic agents for AD treatment. Hybrid compounds such as huperzine A–tacrine hybrids [5], donepezil–tacrine hybrid related derivatives [6–7] and tacrine–indole hybrids [8] with dual-binding site properties exhibit potent AChE inhibition activities. In addition, coumarin compounds linked with various side chain moieties [9–11] as well as with benzylpyridinium moieties [12–14] have been reported as dual-binding site AChE inhibitors. Recently we have reported the AChE inhibitory activity of the coumarin derivative, scopoletin conjugated with a pyridinium side chain (Figure 1) [15] and a docking study revealed that the scopoletin portion of the compound binds to amino acid residues in PAS, whereas the *N*-benzylpyridinium moiety binds to those present in the CAS of AChE. To develop potent AChE inhibitors, we were interested in aminocoumarin as a replacement of scopoletin due to the presence of the amino group in the former that may be useful for binding with this enzyme. Herein, we report our progress on the synthesis, biological evaluation, and molecular docking of 3-aminocoumarin linked with the benzylpyridinium moiety through an amide bond.

## Results and Discussion

### Chemistry

**Scheme 1:**
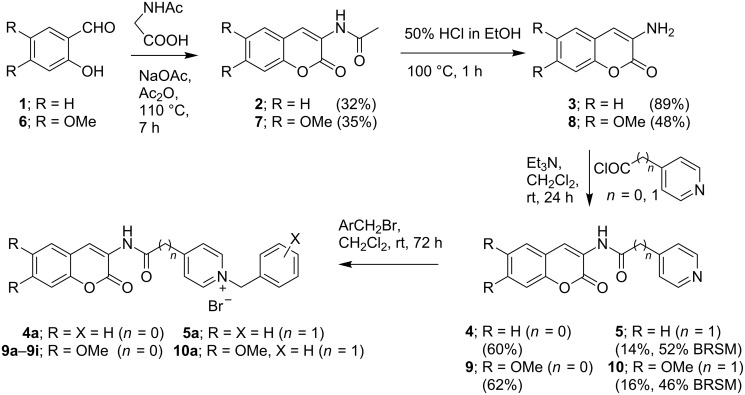
Synthesis of 3-aminocoumarin-*N*-benzylpyridinium salts.

The target 3-aminocoumarin-*N*-benzylpyridinium compounds **4a**, **5a**, **9a**–**9i**, and **10a** were synthesized as shown in Scheme 1. 3-Acetamidocoumarin (**2**) was prepared following Dakin’s procedure [16] by reacting salicylaldehyde (**1**) with *N*-acetylglycine in acetic anhydride at 110 °C for 7 h. Hydrolysis of **2** with 50% HCl in ethanol at 100 °C for 1 h provided 3-aminocoumarin (**3**). Treatment of **3** with either isonicotinyl chloride (*n* = 0) or 4-pyridylacetyl chloride (*n* = 1), generated by reacting the corresponding carboxylic acid with oxalyl chloride, in the presence of triethylamine provided the *N*-acyl-3-aminocoumarins **4** or **5**, respectively. The analogs **9** and **10**, which contain dimethoxy substituents on the coumarin ring, were prepared in the same manner, starting from 2-hydroxy-4,5-dimethoxybenzaldehyde (**6**). Finally, the benzylpyridinium bromide salts **4a**, **5a**, **9a**–**9i**, and **10a** were obtained by treatment of **4**, **5**, **9**, and **10** with substituted benzyl bromides in dichloromethane at room temperature for 72 h.

### Inhibition of AChE

The AChE inhibitory activity of the synthesized compounds was evaluated by Ellman’s method [17] using *Electrophorus electricus* AChE; donepezil hydrochloride and tacrine were used as the reference compounds. The tested compounds with more than 50% enzyme inhibition at a concentration of 1 μM were further evaluated for their half maximal inhibitory concentration (IC_50_) values, and the results are shown in Table 1.

**Table 1:** Acetylcholinesterase inhibitory activity of 3-aminocoumarin derivatives.

structure	compound	R	X	*n*	IC_50_ (nM) ± SD^a,b^

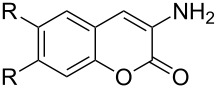	**3**	H	–	–	12%^c^
	**8**	OMe	–	–	5%^c^
				
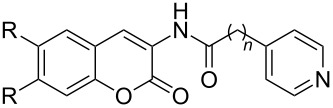	**4**	H	–	0	0%^c^
	**5**	H	–	1	8%^c^
	**9**	OMe	–	0	9%^c^
	**10**	OMe	–	1	9%^c^
				
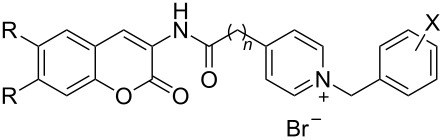	**4a**	H	H	0	71.88 ± 3.44
	**5a**	H	H	1	10%^c^
	**9a**	OMe	H	0	12.48 ± 0.71
	**10a**	OMe	H	1	1087.7 ± 0.05
	**9b**	OMe	2-Cl	0	6.03 ± 0.18
	**9c**	OMe	3-Cl	0	11.47 ± 0.63
	**9d**	OMe	4-Cl	0	293.17 ± 13.57
	**9e**	OMe	2-F	0	3.05 ± 0.28
	**9f**	OMe	3-F	0	5.04 ± 0.26
	**9g**	OMe	4-F	0	5.31 ± 0.38
	**9h**	OMe	2,3-di-F	0	1.53 ± 0.01
	**9i**	OMe	2,6-di-F	0	2.43 ± 0.18
				
	donepezil·HCl	–	–	–	53.51 ± 3.12
	tacrine	–	–	–	190.37 ± 4.55

^a^Concentration of the compound that produced 50% inhibition of enzyme activity. ^b^Results are expressed as mean ± standard error with the average of triplicate independent experiments. ^c^% Inhibition at concentration of 1 μM.

3-Aminocoumarins **3** and **8** were found to be inactive and did not inhibit AChE activity (12% and 5% at 1 μM, respectively). Also, their conversion to *N*-acyl-3-aminocoumarins **4**, **5**, **9**, and **10** did not lead to any improvement (% inhibitions of all compounds were less than 10% at 1 μM). In order to screen the AChE inhibitory activities of the pyridinium salts, compounds **4**, **5**, **9**, and **10** were treated with benzyl bromide to provide the corresponding *N*-benzylpyridinium salts **4a**, **5a**, **9a**, and **10a**, respectively. The conversion of **4** to **4a** resulted in an increased AChE inhibitory activity (from inactive to an IC_50_ of 71.88 ± 3.40 nM). On the other hand, the corresponding *N*-benzylpyridinium salt of **5** (compound **5a**) displayed only poor activity, which was comparable to that of its parent compound (10% inhibition at concentration of 1 μM). A comparison of the IC_50_ values of **4a** and **5a** indicated that the presence of a methylene unit between the carbonyl group and the pyridine moiety lowered the inhibitory potency. This hypothesis was confirmed when comparing the AChE inhibitory activity of **9a** with that of **10a**. The *N*-benzylpyridinium salt **9a**, lacking the aforementioned methylene group possessed an IC_50_ value against AChE of 12.48 ± 0.71 nM, whereas the *N*-benzylpyridinium salt **10a**, obtained from **10**, had an IC_50_ value of 1087.7 ± 0.05 nM. Based on these findings, the coumarin isonicotinamides **4** or **9** were selected as appropriate precursors for the preparation of the subsequent pyridinium salts. The presence of methoxy groups in the 6 and 7 positions of the coumarin ring remarkably enhanced the inhibitory activity against AChE as observed from the comparisons of compounds **9a** (IC_50_ of 12.48 ± 0.71 nM) and **4a** (IC_50_ of 71.88 ± 3.44 nM), and of compounds **10a** (IC_50_ of 1087.7 ± 0.05 nM) and **5a** (10% at a concentration of 1 μM). The results suggested that combining the *N*-benzylpyridinium moiety with the 3-amino-6,7-dimethoxycoumarin core through the amide bond provides a significant enhancement of the AChE inhibitory activity. As mentioned above, compound **9** was then selected as the parent core for the synthesis of other substituted benzylpyridinium salts for further evaluation of their AChE inhibitory activity.

On the basis of the potent activity of **9a** (IC_50_ of 12.48 ± 0.71 nM), further modifications by introducing chlorine or fluorine substituents at the *ortho*-, *meta*-, or *para-*positions on the benzyl group were investigated. The results showed that the type and the position of the substituents on the benzyl group influenced the inhibitory activity against AChE. For chlorine as a substituent, the activity followed the order of *ortho* > *meta* > *para.* Compared with benzylpyridinium salt **9a**, a chlorine substitution at the *o*-position (**9b**) provided a two-fold improvement in activity, whereas *m-* and *p*-substitutions (**9c** and **9d**, respectively) led to comparable and diminished inhibitory activities, respectively. For fluorine as a substituent, the activity also followed the order of *ortho* > *meta* ≈ *para*. The AChE inhibitory activities of all fluorine analogs were superior to those of the chlorine analogs and the unsubstituted benzylpyridinium salt **9a**. In detail, a fluorine substituent at the *ortho*-position (**9e**) increased the inhibitory activity with an IC_50_ value of 3.05 ± 0.28 nM, whereas the *m-* and *p*-fluoro-substituted analogs **9f** and **9g** provided comparable IC_50_ values of 5.04 ± 0.26 and 5.31 ± 0.38 nM, respectively. Difluorinated benzylpyridinium salts were also examined for their inhibitory activities and were found to be more active than the corresponding monofluorinated compounds. Thus compound **9h**, a 2,3-difluorobenzylpyridinium salt, exhibited the highest activity in this study with an IC_50_ value of 1.53 ± 0.01 nM and compound **9i** having a 2,6-difluorobenzyl substituent showed a slightly inferior activity with an IC_50_ value of 2.43 ± 0.18 nM. The IC_50_ value of **9h**, the most active compound in this study, was 35-fold lower than that of donepezil hydrochloride (IC_50_ of 53.51 ± 3.12 nM) and 124-fold lower than that of tacrine (IC_50_ of 190.37 ± 4.55 nM). Moreover, **9h** was inactive against MRC-5 normal embryonic lung cell (0% cytotoxicity at concentration of 50 μg/mL).

### Molecular docking studies

Molecular docking studies [15] were performed to study the binding mode and interactions of the synthesized compounds with AChE. A crystal structure of recombinant human acetylcholinesterase complexed with donepezil (PDB code 4ey7) [18] retrieved from the Protein Data Bank was used in the study because of its high similarity (approximately 88% similarity), good resolution (at 2.35 Å) and ligand state with donepezil of Human AChE structure. Molecular docking of AChE via the GOLD v5.2.2 program [19] was used to investigate the orientation of the compounds in the rhAChE binding site. Compounds **4a**, **9a**, **9b**, **9e**, **9h**, **9i**, and **10a** were selected as representatives for the docking study.

The active site of human AChE, which is at the bottom of a deep and narrow gorge, consists of several major domains. A catalytic triad (CT) that comprises Ser203, His447, and Glu334 residues is responsible for hydrolyzing the ester bond of ACh [20]. The CAS, which is responsible for the binding of the quaternary ammonium moiety of choline, consists of Trp86, Tyr133, Tyr337, and Phe338 [21]. The acyl pocket, which binds to the acetyl group of ACh, consists of Phe295 and Phe297 residues [21]. The oxyanion hole that interacts with the ACh carbonyl oxygen consists of Gly121, Gly122, and Ala204 [22–23]. In addition to the active site another binding site, known as PAS, is located around the entrance of the active site gorge. The PAS consists of Tyr72, Asp74, Tyr124, Trp286, and Tyr341 [23–24].

**Figure 2:**
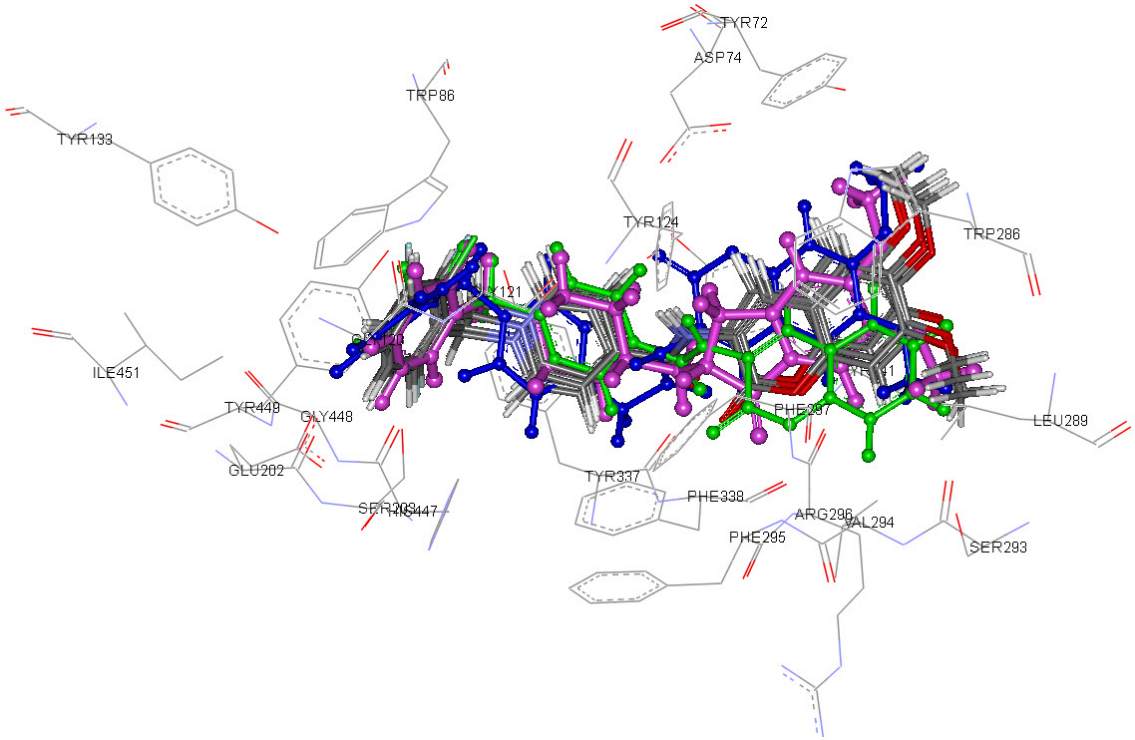
Docked conformations of donepezil (ball-and-stick model; pink), compounds **9a**, **9b**, **9e**, **9h**, and **9i** (stick model; colored atom-type), compound **4a** (ball-and-stick model; green), and compound **10a** (ball-and-stick model; blue).

From the docking result, the docked orientation of donepezil showed a root-mean-square deviation (RMSD) of 0.474 Å when compared with the conformation in the crystal structure, thereby indicating its potential as a suitable method for the binding mode of compounds in the rhAChE binding site. The docked conformations of donepezil and the synthesized compounds are shown in Figure 2. All docked compounds were located similar to donepezil in the binding pocket. The benzylpyridinium moiety was bound close to the CT and the CAS while the chromene rings were located at the PAS. Compounds **9a**, **9b**, **9e**, **9h**, and **9i** revealed similar orientations in the binding pocket whereas the binding of compounds **4a** and **10a** was partially different. The orientation of the chromene ring of compound **4a** was shifted away from the others, whereas the chromene ring of compound **10a** was flipped to the opposite site when compared with the other compounds.

**Figure 3:**
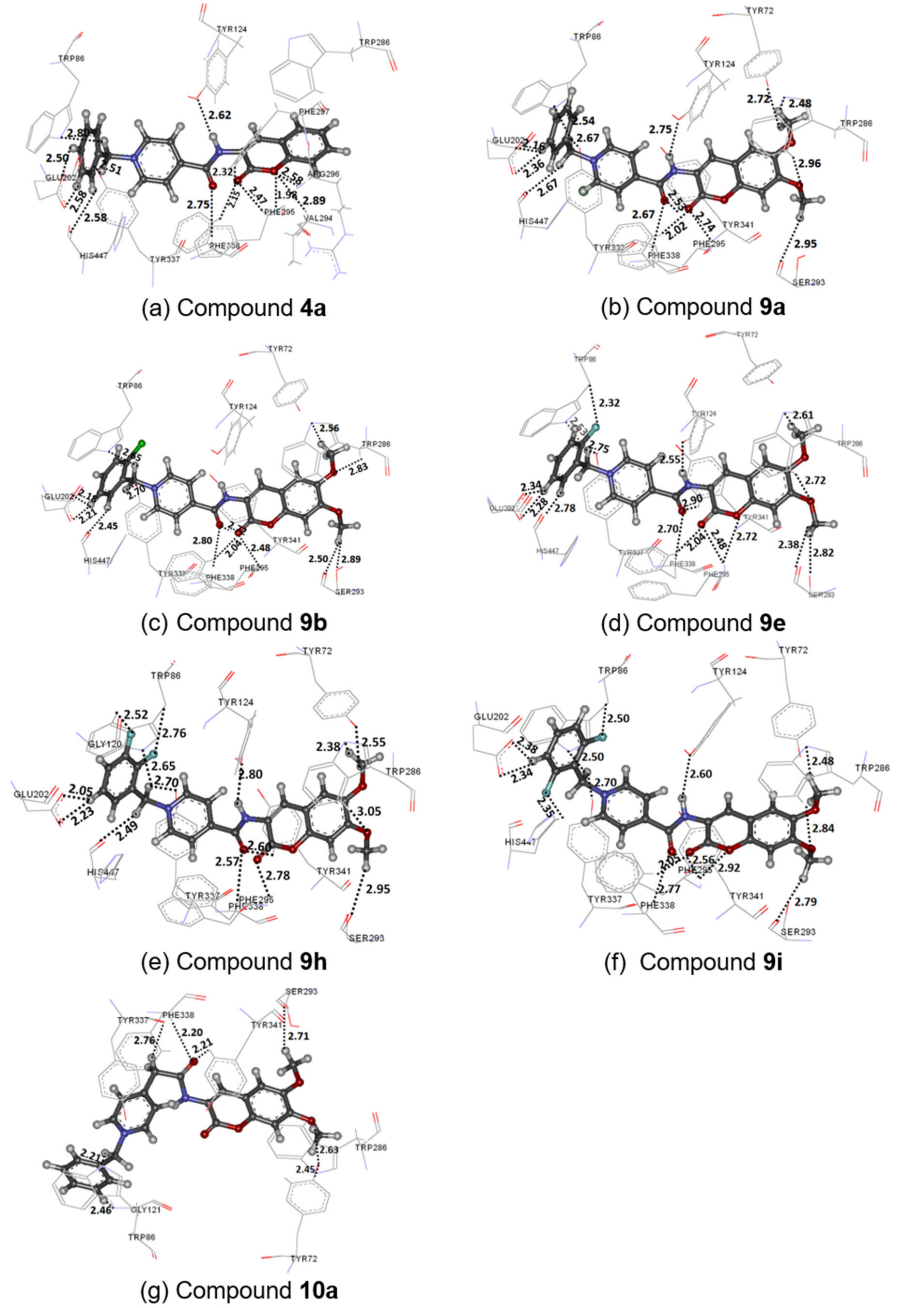
Binding interactions in the rhAChE binding pocket with (a) **4a**, (b) **9a**, (c) **9b**, (d) **9e**, (e) **9h**, (f) **9i**, and (g) **10a**. Distances are shown in angstrom (Å).

The binding interactions of each compound are shown in Figure 3. The most active compound **9h** (Figure 3e) formed H-bond interactions to the residues in the following binding sites: (1) the methylene unit of the benzyl group to the CT amino acid residue His447; (2) the carbonyl of the amide moiety and fluorine atoms to the amino acid residues Trp86, Tyr337, and Phe338 in the CAS; (3) the carbonyl group of the chromene ring to the acyl pocket amino acid residue Phe295; (4) the nitrogen atom of the amide group and dimethoxy substituents on the chromene ring to the amino acid residues Tyr72, Tyr124, Trp286, and Tyr341 in the PAS; and (5) the hydrogen atoms of phenyl ring to Glu202 and a methoxy group to Ser293. Moreover, C–H–π and π–π interactions were found for compound **9h**. The C–H–π interactions were found between hydrogen atoms of the methoxy group to the PAS amino acid residue Tyr72 and the pyridinium ring to the CAS amino acid residue Phe338, whereas π–π interactions were found among the chromene ring, pyridinium, and phenyl ring to Trp286, Phe338, and Trp86, respectively. When the position of the fluoro-substituent(s) of **9h** was changed or removed, the inhibitory activity decreased, as observed in the cases of compounds **9e** (Figure 3d) and **9i** (Figure 3f). From the docked conformation of compounds **9e** and **9i**, the absence of the 3-fluoro-substituent caused a loss of the H-bond interaction to Trp86 in the CAS. Their conformations were slightly shifted up from Trp86 to form stronger a H-bond interaction between the 2-fluoro-substituent and a hydrogen atom of the β-carbon, thereby causing the slight shift of the remaining parts of the structures. For instance, the methoxy group of the chromene ring moved away from Tyr72 in the PAS; thus, compounds **9e** and **9i** lost H-bond interactions to Tyr72. Moreover, the C–H–π interactions of compounds **9e** and **9i** to Tyr72 became weaker. All other H-bonds, C–H–π and π–π interactions of **9e** and **9i** to amino acid residues in the binding site were similar to those found in compound **9h**. When comparing the interaction between compounds **9e** and **9i**, the 6-fluoro-substituent in compound **9i** can form a stronger H-bond interaction to His447. The absence of the 6-fluoro-substituent also caused a slight shift of the methoxy position on the chromene ring away from Tyr72. Therefore, this phenomenon can be a reason for the inferior activity of compound **9e** when compared with compound **9i**. For compound **9b** (Figure 3c), the presence of a bulkier chlorine substituent at the *o*-position of the phenyl ring resulted in the shift of the phenyl ring away from Trp86, as well as shifting of other parts. These shifts led to slightly weaker C–H–π and π–π interactions between compound **9b** and Tyr72, Trp86, Tyr337, and Phe338. In case of compound **9a** (Figure 3b), the docked conformation was aligned similar to the conformation of compound **9h**. However, because of the absence of fluoro-substituents, compound **9a** does not form a H-bond interaction to Trp86, which explains the lower activity of compound **9a** compared with compound **9h**. For compounds **4a** (Figure 3a) and **10a** (Figure 3g), the docked conformations were partially different from the other compounds. In the case of **4a**, having no methoxy substituents on the chromene ring, no H-bonds and C–H–π interactions to Tyr72, Trp286, and Tyr341 are observed. In compound **10a**, one additional methylene group between the pyridinium and the carbonyl groups caused the chromene ring to flip in the binding pocket. The other parts of the structure also shifted when compared with the other compounds. Compared with compound **9h**, these shifts of docked compound **10a** led to the loss of H-bonds, as well as C–H–π and π–π interactions with AChE, thereby reducing the inhibitory activity.

The docking study showed that the docked orientations of the selected compounds in the binding pocket were similar to that of donepezil. The results indicated binding interactions between the chromene ring and the PAS amino acids residues as well as between the benzylpyridinium moiety and the CAS amino acid residues. Interactions between the carbonyl groups of the chromene ring and of the amide group to Phe338 in the CAS and between the nitrogen atom of the amide group to Tyr124 in the PAS were the key interactions of the synthesized 3-aminocoumarin-*N*-benzylpyridinium conjugates in the binding pocket of AChE.

## Conclusion

A series of 3-amino-6,7-dimethoxycoumarins conjugated with the *N*-benzylpyridinium moiety through an amide linkage was synthesized and investigated for their AChE inhibitory activities. A number of benzylpyridinium derivatives exhibited potent activity with IC_50_ values in the nanomolar concentration range. The results showed that the dimethoxy substituent on the chromene ring, the length of the amide linker, as well as the type and position of substituents on the benzyl group all contributed significantly to the AChE inhibitory activity. The 6,7-dimethoxy-substituted chromene ring significantly enhances the activity. An additional methylene unit of the linker between the amide carbonyl group and the pyridine ring led to a diminished activity. The fluorine substituent at the *ortho*-position on the benzyl ring provided a remarkable increase in AChE inhibition. Among the derivatives, the 2,3-difluorobenzylpyridinium compound was the most potent with an IC_50_ value of 1.53 ± 0.01 nM, followed by the 2,6-difluorobenzylpyridinium and 2-fluorobenzylpyridinium analogs with IC_50_ values of 2.43 ± 0.18 and 3.05 ± 0.28 nM, respectively. Docking studies revealed that the synthesized compounds can act as dual binding site inhibitors by allowing the coumarin portion to bind with the PAS and the *N*-benzylpyridinium residue to bind with the CAS of AChE.

## Supporting Information

File 1 Experimental details, characterization data and copies of NMR spectra.
