# Supplementary material for: Synthesis of 3-aminocoumarin-N-benzylpyridinium conjugates with nanomolar inhibitory activity against acetylcholinesterase
Source: Beilstein J Org Chem. 2018 Oct 2;14:2545–52. doi: 10.3762/bjoc.14.231 (PMC6204823; doi:10.3762/bjoc.14.231)

Supporting Information
for
Synthesis of 3-aminocoumarin-*N*-benzylpyridinium conjugates with nanomolar inhibitory activity against acetylcholinesterase

Nisachon Khunnawutmanotham[1], Cherdchai Laongthipparos[2], Patchreenart Saparpakorn[3], Nitirat Chimnoi[4], and Supanna Techasakul[*1]

Address: [1]Laboratory of Organic Synthesis, Chulabhorn Research Institute, 54 Kamphaeng Phet 6, Talat Bang Khen, Lak Si, Bangkok 10210, Thailand, [2]Department of Industrial Chemistry, Faculty of Applied Science, King Mongkut's University of Technology North Bangkok, Bangkok 10800, Thailand, [3]Department of Chemistry, Faculty of Science, Kasetsart University, Bangkok 10900, Thailand, and [4]Laboratory of Natural Products, Chulabhorn Research Institute, 54 Kamphaeng Phet 6, Talat Bang Khen, Lak Si, Bangkok 10210, Thailand

Email: Supanna Techasakul - supanna@cri.or.th
*Corresponding author

Experimental details, characterization data and copies of NMR spectra

Experimental

Chemistry

^{1}H and ^{13}C NMR spectra were recorded on Bruker Avance 300, Bruker Avance 400, and Bruker Avance 600 spectrometers. Chemical shifts were reported as δ values in ppm relative to tetramethylsilane. Coupling constants (*J*) are given in Hz. Mass spectra were obtained on a Finnigan POLARIS Ion Trap Mass Spectrometer and accurate masses (HRMS) were recorded on a Bruker MicroTOF Mass Spectrometer. The infrared (IR) spectra were determined in cm^{-1} with a Perkin Elmer Spectrum One FT-IR spectrometer. Column chromatography was performed on Merck silica gel 60 (70–230 mesh).

***N*-(2-Oxo-2*H*-chromen-3-yl)acetamide (2).** A mixture of salicylaldehyde (5.0 g, 0.04 mol), *N*-acetylglycine (4.8 g, 0.04 mol), anhydrous sodium acetate (13.4 g, 0.16 mol), and acetic anhydride (19.3 mL, 0.20 mol) was heated at 110 °C for 7 h. After cooling to ambient temperature, cold water was added to the reaction mixture and the precipitates collected and washed with water. Recrystallization from ethanol yielded **2** (2.3 g, 28%) as white solid. ^{1}H-NMR (DMSO-d_6, 300 MHz), *δ*: 9.75 (s, 1H), 8.58 (s, 1H), 7.67 (d, *J* = 7.5 Hz, 1H), 7.50–7.40 (m, 1H), 7.40–7.30 (m, 2H), 2.16 (s, 3H); ^{13}C-NMR (DMSO-d_6, 75 MHz), *δ*: 170.2, 157.5, 149.6, 129.5, 127.8, 124.9, 124.5, 123.5, 119.6, 115.8, 23.9; HRMS (ESI-pos), *m/z* calcd. for $C_{11}H_9NO_3Na$ $[M + Na]^+$: 226.0475, measured: 226.0482.

The preparation of *N*-(2-oxo-2*H*-chromen-3-yl)acetamide (**2**) was previously described [1].

3-Amino-2*H*-chromen-2-one (3). A mixture of **2** (2.04 g, 0.01 mol) in 50% hydrochloric acid/ethanol was heated at 100 °C for 1 h. After cooling to ambient temperature, the reaction mixture was concentrated under reduced pressure to remove ethanol. The residue was dissolved in water, and 2 N NaOH was added to adjust the pH to 8. The precipitates were collected and washed with water to yield **3** (1.45 g, 90%) as pale yellow solid. ^{1}H-NMR ($CDCl_3$, 300 MHz), *δ:* 7.40–7.15 (m, 4H), 6.70 (s, 1H), 4.29 (s, 1H); ^{13}C-NMR ($CDCl_3$, 75 MHz), *δ*: 159.4, 149.0, 132.0, 126.6, 125.0, 124.6, 121.1, 116.1, 110.8; HRMS (ESI-pos), *m/z* calcd. for $C_9H_7NO_2Na$ $[M+Na]^+$: 184.0369, measured: 184.0370.

The preparation of 3-amino-2*H*-chromen-2-one (**3**) was previously described [1,2] and ^{1}H NMR data for **3** were in agreement with that previously reported [2].

***N*-(6,7-Dimethoxy-2-oxo-2*H*-chromen-3-yl)acetamide (7).** A mixture of **6** (1.0 g, 5.45 mmol), *N*-acetylglycine (0.64 g, 5.45 mmol), anhydrous sodium acetate (1.8 g, 21.9 mmol), and acetic anhydride (2.6 mL, 27.55 mmol) was heated at 110 °C for 7 h. After cooling to ambient temperature, the reaction mixture was diluted with water and extracted with ethyl acetate. The combined organic layer was washed with water, dried over anhydrous sodium sulfate, filtered, and then concentrated under reduced pressure. The residue was purified by column chromatography to yield **7** (0.5 g, 35%) as white solid. ^{1}H-NMR ($CDCl_3$, 300 MHz), *δ*: 8.62 (s, 1H), 8.01 (br s, 1H), 6.89 (s, 1H), 6.83 (s, 1H), 3.94 (s, 3H), 3.92 (s, 3H), 2.24 (s, 3H); ^{13}C-NMR ($CDCl_3$, 75 MHz), *δ*: 169.2, 159.0, 151.2, 147.0, 145.3, 123.9, 122.1, 112.2, 108.0, 99.7, 56.3(2C), 24.7; FT-IR, ν_{max} (cm^{-1}): 3347, 1715, 1682, 1530, 1386, 1244, 1010;

MS (EI), *m/z* (relative intensity): 263 (M^+, 47), 221 (100), 206 (39), 178 (17), 150 (16), 122 (26); HRMS (ESI-pos), *m/z* calcd. for $C_{13}H_{13}NO_5Na$ $[M + Na]^+$: 286.0686, measured: 286.0690.

The preparation and ^{1}H NMR data for *N*-(6,7-dimethoxy-2-oxo-2*H*-chromen-3-yl)acetamide (**7**) were previously described [3].

3-Amino-6,7-dimethoxy-2*H*-chromen-2-one (8). A mixture of **7** (380 mg, 1.44 mmol) in 50% hydrochloric acid/ethanol was heated at 100 °C for 1 h. After cooling to ambient temperature, the reaction mixture was concentrated under reduced pressure to remove ethanol. The residue was dissolved in water and 2 N NaOH was added to adjust the pH to 8. The precipitates were collected and washed with water to yield **8** (155 mg, 48%) as pale yellow solid. ^{1}H-NMR ($CDCl_3$, 300 MHz), *δ:* 6.82 (s, 1H), 6.70 (s, 1H), 6.66 (s, 1H), 3.90 (s, 3H), 3.89 (s, 3H); ^{13}C-NMR ($CDCl_3$, 75 MHz), *δ*: 159.7, 148.9, 146.6, 144.0, 130.3, 113.5, 112.1, 106.2, 100.0, 56.3, 56.2; FT-IR, ν_{max} (cm^{-1}): 3317, 1711, 1697, 1290, 1152, 1004; MS (EI), *m/z* (relative intensity): 221 (M^+, 44), 206 (11), 146 (63), 99 (68), 70 (23), 61 (37), 56 (100); HRMS (ESI-pos), *m/z* calcd. for $C_{11}H_{12}NO_4$ $[M+H]^+$: 222.0761, measured: 222.0760.

General procedure for the synthesis of *N*-acyl-3-aminocoumarins.

A stirred solution of carboxylic acid (1.2 equiv) in dichloromethane (10 mL) was treated with oxalyl chloride (1.44 equiv) followed by a catalytic amount of *N*,*N*-dimethylformamide, and the resulting mixture was stirred at room temperature for 1 h. Then, the solvent was removed under reduced pressure to afford the acid

chloride. This acid chloride was redissolved in dichloromethane (10 mL) and added dropwise to a solution of 3-aminocoumarin (1 equiv) in dichloromethane (10 mL) in the presence of triethylamine (2.4 equiv). After stirring at room temperature for 24 h, the mixture was poured into a solution of saturated aqueous sodium hydrogen carbonate and extracted with dichloromethane. The combined organic layer was washed with water, dried over anhydrous sodium sulfate, filtered, and then concentrated under reduced pressure. The residue was recrystallized from ethanol to yield *N*-acyl-3-aminocoumarin.

***N*-(2-Oxo-2*H*-chromen-3-yl)isonicotinamide (4).** Prepared from **3** and isonicotinic acid, **4** was obtained as white solid (60%). ^{1}H-NMR (DMSO-d_6, 300 MHz), δ: 10.12 (s, 1H), 8.81 (d, J = 6.0 Hz, 2H), 8.64 (s, 1H), 7.86 (d, J = 6.0 Hz, 2H), 7.81 (d, J = 9.0 Hz, 1H), 7.59 (t, J = 6.0 Hz, 1H), 7.46 (d, J = 6.0 Hz, 1H), 7.40 (t, J = 9.0 Hz, 1H); ^{13}C-NMR (DMSO-d_6, 75 MHz), δ: 164.8, 157.5, 150.4(2C), 150.3, 140.6, 130.6, 128.4, 128.3, 125.1, 123.7, 121.5(2C), 119.1, 116.0; FT-IR, v_{max} (cm^{-1}): 3376, 1709, 1668, 1543, 1369, 747; MS (EI), *m/z* (relative intensity): 266 (M$^+$, 58), 238 (4), 132 (3), 106 (100), 78 (29); HRMS (ESI-pos), *m/z* calcd. for $C_{15}H_{11}N_2O_3$ [M + H]$^+$: 267.0764, measured: 267.0773.

***N*-(2-Oxo-2*H*-chromen-3-yl)-2-(pyridin-4-yl)acetamide (5).** Prepared from **3** and 4-pyridylacetic acid hydrochloride, **5** was obtained as pale yellow solid (14%, 52% BRSM). ^{1}H-NMR ($CDCl_3$, 300 MHz), δ: 8.68 (s, 1H), 8.63 (d, J = 6.0 Hz, 2H), 8.26 (br s, 1H), 7.50–7.43 (m, 2H), 7.34–7.27 (m, 4H), 3.79 (s, 2H); ^{13}C-NMR ($CDCl_3$, 75 MHz), δ: 168.3, 158.6, 150.3, 150.0(2C), 142.5, 130.0, 127.9, 125.3, 124.4(2C), 124.0, 123.7, 119.6, 116.4, 43.7; FT-IR, v_{max} (cm^{-1}): 3323, 1713, 1688, 1535, 1365,

759; MS (EI), *m/z* (relative intensity): 280 (M^+, 22), 161 (100), 133 (29), 120 (67), 92 (21); HRMS (ESI-pos), *m/z* calcd. for $C_{16}H_{13}N_2O_3$ $[M + H]^+$: 281.0921, measured: 281.0925.

***N*-(6,7-Dimethoxy-2-oxo-2*H*-chromen-3-yl)isonicotinamide (9).** Prepared from **8** and isonicotinic acid, **9** was obtained as pale yellow solid (62%). ^{1}H-NMR (DMSO-d_6, 300 MHz), δ: 10.00 (s, 1H), 8.80 (d, J = 6.0 Hz, 2H), 8.54 (s, 1H), 7.85 (d, J = 6.0 Hz, 2H), 7.36 (s, 1H), 7.14 (s, 1H), 3.88 (s, 3H), 3.83 (s, 3H); ^{13}C-NMR (DMSO-d_6, 75 MHz), δ: 164.6, 158.0, 151.6, 150.3(2C), 146.4, 146.2, 140.7, 130.2, 121.4(2C), 121.0, 111.2, 109.0, 99.9, 56.2, 56.0; FT-IR, v_{max} (cm^{-1}): 3325, 1710, 1665, 1535, 1385, 1248, 1006, 850; MS (EI), *m/z* (relative intensity): 326 (M^+, 100), 298 (6), 192 (55), 164 (5), 106 (30), 78 (5); HRMS (ESI-pos), *m/z* calcd. for $C_{17}H_{15}N_2O_5$ $[M+H]^+$: 327.0975, measured: 327.0983.

***N*-(6,7-Dimethoxy-2-oxo-2*H*-chromen-3-yl)-2-(pyridin-4-yl)acetamide (10).** Prepared from **8** and 4-pyridylacetic acid hydrochloride, **10** was obtained as pale yellow solid (16%, 46% BRSM). ^{1}H-NMR (DMSO-d_6, 300 MHz), δ: 9.95 (s, 1H), 8.57 (s, 1H), 8.51 (d, J = 6.0 Hz, 2H), 7.36 (d, J = 6.0 Hz, 2H), 7.26 (s, 1H), 7.07 (s, 1H), 3.90 (s, 2H), 3.85 (s, 3H), 3.79 (s, 3H); ^{13}C-NMR (DMSO-d_6, 75 MHz), δ: 169.3, 157.8, 150.9, 149.4(2C), 146.3, 145.2, 144.6, 125.9, 124.6(2C), 121.8, 111.5, 108.8, 99.8, 56.1, 55.9, 41.8; FT-IR, v_{max} (cm^{-1}): 3371, 1695, 1677, 1531, 1386, 1229, 1003; MS (EI), *m/z* (relative intensity): 340 (M^+, 89), 221 (100), 206 (34), 192 (11), 178 (8), 92 (8); HRMS (ESI-pos), *m/z* calcd. for $C_{18}H_{17}N_2O_5$ $[M+H]^+$: 341.1132, measured: 341.1129.

General procedure for the synthesis of *N*-benzylpyridinium salts.

A solution of **4**, **5**, **9**, **10** (1 equvi), and the appropriate benzyl bromide (3 equiv) in dichloromethane was stirred at room temperature for 72 h. The precipitates were then filtered and washed with dichloromethane to give the solid pyridinium salts.

1-Benzyl-4-(2-oxo-2*H*-chromen-3-ylcarbamoyl)pyridinium bromide (4a).

Prepared from **4** and benzyl bromide, **4a** was obtained as yellow solid (75%). ^{1}H-NMR (DMSO-d_6, 300 MHz), δ: 10.77 (s, 1H), 9.45 (d, J = 6.0 Hz, 2H), 8.70 (s, 1H), 8.52 (d, J = 6.0 Hz, 2H), 7.84 (d, J = 9.0 Hz, 1H), 7.65–7.35 (m, 8H), 5.99 (s, 2H); ^{13}C-NMR (DMSO-d_6, 75 MHz), δ: 163.4, 157.7, 151.0, 149.0, 146.1(2C), 134.7, 131.3, 130.0, 129.7(2C), 129.3(2C), 129.0, 128.8, 127.4(2C), 125.7, 124.1, 119.4, 116.5, 63.9; FT-IR, ν_{max} (cm^{-1}): 3459, 3397, 1722, 1666, 1547, 1455, 1371, 752, 699; MS (EI), *m/z* (relative intensity): 266 ([M-CH_2Ph]$^{+}$, 56), 238 (6), 129 (10), 106 (100), 91 (86), 78 (28); HRMS (ESI-pos), *m/z* calcd. for $C_{22}H_{17}N_2O_3$ [M]$^{+}$: 357.1234, measured: 357.1246.

1-Benzyl-4-((2-oxo-2*H*-chromen-3-ylcarbamoyl)methyl)pyridinium bromide (5a).

Prepared from **5** and benzyl bromide, **5a** was obtained as yellow solid (90%). ^{1}H-NMR (DMSO-d_6, 300 MHz), δ: 10.30 (s, 1H), 9.20 (d, J = 6.6 Hz, 2H), 8.58 (s, 1H), 8.13 (d, J = 6.6 Hz, 2H), 7.67 (dd, J = 7.8, 1.5 Hz, 1H), 7.57–7.31 (m, 8H), 5.87 (s, 2H), 4.30 (s, 2H); ^{13}C-NMR (DMSO-d_6, 75 MHz), δ: 168.0, 157.4, 155.5, 149.8, 144.1(2C), 134.3, 129.9, 129.3, 129.2(2C), 129.1(2C), 128.8(2C), 127.9, 125.0, 124.7, 124.1, 119.2, 115.8, 62.7, 42.0; FT-IR, ν_{max} (cm^{-1}): 3185, 1717, 1683, 1517, 1363, 1175, 750, 733, 704; MS (EI), *m/z* (relative intensity): 280 ([M-CH_2Ph]$^{+}$, 6), 236

(5), 210 (16), 187 (23), 161 (100), 133 (41), 91 (24), 78 (21); HRMS (ESI-pos), *m/z* calcd. for $C_{23}H_{19}N_2O_3$ $[M]^+$: 371.1390, measured: 371.1403.

1-Benzyl-4-(6,7-dimethoxy-2-oxo-2*H*-chromen-3-ylcarbamoyl)pyridinium bromide (9a). Prepared from **9** and benzyl bromide, **9a** was obtained as yellow solid (56%). ^{1}H-NMR (DMSO-d_6, 300 MHz), δ: 10.64 (s, 1H), 9.44 (d, J = 9.0 Hz, 2H), 8.62 (s, 1H), 8.52 (d, J = 9.0 Hz, 2H), 7.61–7.44 (m, 5H), 7.39 (s, 1H), 7.12 (s, 1H), 5.98 (s, 2H), 3.87 (s, 3H), 3.82 (s, 3H); ^{13}C-NMR (DMSO-d_6, 75 MHz), δ: 162.5, 157.6, 151.8, 148.5, 146.4, 146.3, 145.6(2C), 134.2, 130.0, 129.4, 129.2(2C), 128.8(2C), 126.8(2C), 128.0, 111.0, 109.1, 99.9, 63.4, 56.2, 56.0; FT-IR, v_{max} (cm^{-1}): 3335, 1723, 1674, 1536, 1384, 1245, 990; MS (EI), *m/z* (relative intensity): 326 ([M-$CH_2Ph]^+$, 40), 192 (26), 164 (100), 151 (35), 106 (15), 91 (22); HRMS (ESI-pos), *m/z* calcd. for $C_{24}H_{21}N_2O_5$ $[M]^+$: 417.1445, measured: 417.1456.

1-Benzyl-4-((6,7-dimethoxy-2-oxo-2*H*-chromen-3-ylcarbamoyl)methyl)pyridinium bromide (10a). Prepared from **10** and benzyl bromide, **10a** was obtained as yellow solid (47%). ^{1}H-NMR (DMSO-d_6, 300 MHz), δ: 10.17 (s, 1H), 9.15 (d, J = 9.0 Hz, 2H), 8.54 (s, 1H), 8.12 (d, J = 9.0 Hz, 2H), 7.55–7.44 (m, 5H), 7.24 (s, 1H), 7.08 (s, 1H), 5.84 (s, 2H), 4.26 (s, 2H), 3.85 (s, 3H), 3.78 (s, 3H); ^{13}C-NMR (DMSO-d_6, 75 MHz), δ: 168.1, 158.2, 156.2, 151.6, 146.9, 145.9, 144.6(2C), 134.8, 129.9, 129.7(2C), 129.6(2C), 129.2(2C), 126.8, 122.1, 111.9, 109.3, 100.3, 63.3, 56.6, 56.4, 42.5; FT-IR, v_{max} (cm^{-1}): 3370, 1709, 1681, 1533, 1383, 1276; MS (EI), *m/z* (relative intensity): 431 ($[M]^+$, 2), 340 (8), 247 (32), 221 (100), 206 (18), 91 (27); HRMS (ESI-pos), *m/z* calcd. for $C_{25}H_{23}N_2O_5$ $[M]^+$: 431.1602, measured: 431.1603.

1-(2-Chlorobenzyl)-4-(6,7-dimethoxy-2-oxo-2*H*-chromen-3-ylcarbamoyl)pyridinium bromide (9b). Prepared from **9** and 2-chlorobenzyl bromide, **9b** was obtained as yellow solid (41%). ^{1}H-NMR (DMSO-d_6, 400 MHz), δ: 10.71 (s, 1H), 9.31 (d, J = 6.7 Hz, 2H), 8.64 (s, 1H), 8.53 (d, J = 6.7 Hz, 2H), 7.63 (d, J = 7.6 Hz, 1H), 7.56–7.47 (m, 3H), 7.40 (s, 1H), 7.14 (s, 1H), 6.11 (s, 2H), 3.87 (s, 3H), 3.83 (s, 3H); ^{13}C-NMR (DMSO-d_6, 100 MHz), δ: 163.5, 158.6, 152.8, 149.8, 147.4, 147.2, 146.9(2C), 134.1, 132.4, 132.3, 132.1, 130.9, 130.8, 129.0, 127.5(2C), 121.6, 111.7, 109.7, 100.5, 61.8, 56.6, 56.3; FT-IR, ν_{max} (cm^{-1}): 3338, 1721, 1675, 1544, 1511, 1387, 1247, 993; MS (EI), *m/z* (relative intensity): 326 ([M- $CH_2C_6H_4Cl]^+$, 81), 192 (37), 125 (100), 106 (32), 97 (12), 57 (13); HRMS (ESI-pos), *m/z* calcd. for $C_{24}H_{20}N_2O_5Cl$ $[M]^+$: 451.1055, measured: 451.1043.

1-(3-Chlorobenzyl)-4-(6,7-dimethoxy-2-oxo-2*H*-chromen-3-ylcarbamoyl)pyridinium bromide (9c). Prepared from **9** and 3-chlorobenzyl bromide, **9c** was obtained as yellow solid (35%). ^{1}H-NMR (DMSO-d_6, 400 MHz), δ: 10.65 (s, 1H), 9.45 (d, J = 6.0 Hz, 2H), 8.62 (s, 1H), 8.52 (d, J = 6.0 Hz, 2H), 7.76 (s, 1H), 7.60–7.48 (m, 3H), 7.40 (s, 1H), 7.13 (s, 1H), 5.97 (s, 2H), 3.87 (s, 3H), 3.82 (s, 3H); ^{13}C-NMR (DMSO-d_6, 100 MHz), δ: 163.0, 158.1, 152.3, 149.1, 147.0, 146.8, 146.2(2C), 136.8, 134.2, 131.6, 130.5, 130.0, 129.4, 128.2, 127.4(2C), 121.3, 111.5, 109.6, 100.4, 63.0, 56.7, 56.5; FT-IR, ν_{max} (cm^{-1}): 3361, 1707, 1675, 1543, 1387, 1249, 997; MS (EI), *m/z* (relative intensity): 326 ([M- $CH_2C_6H_4Cl]^+$, 100), 192 (40), 125 (33), 106 (21), 83 (7), 73 (12); HRMS (ESI-pos), *m/z* calcd. for $C_{24}H_{20}N_2O_5Cl$ $[M]^+$: 451.1055, measured: 451.1063.

1-(4-Chlorobenzyl)-4-(6,7-dimethoxy-2-oxo-2*H*-chromen-3-ylcarbamoyl)pyridinium bromide (9d). Prepared from **9** and 4-chlorobenzyl bromide, **9d** was obtained as yellow solid (20%). ^{1}H-NMR (DMSO-d_6, 600 MHz), δ: 10.62 (s, 1H), 9.36 (d, J = 6.5 Hz, 2H), 8.62 (s, 1H), 8.50 (d, J = 6.5 Hz, 2H), 7.60 (d, J = 8.5 Hz, 2H), 7.56 (d, J = 8.5 Hz, 2H), 7.39 (s, 1H), 7.13 (s, 1H), 5.92 (s, 2H), 3.86 (s, 3H), 3.82 (s, 3H); ^{13}C-NMR (DMSO-d_6, 150 MHz), δ: 162.3, 157.5, 151.9, 148.6, 146.5, 146.3, 145.5(2C), 134.3, 132.8, 130.8(2C), 130.0, 129.1(2C), 126.7(2C), 120.7, 111.0, 109.2, 99.9, 62.6, 56.1, 56.0; FT-IR, ν_{max} (cm^{-1}): 3295, 1698, 1672, 1539, 1513, 1386, 1247; MS (EI), *m/z* (relative intensity): 326 ([M- $CH_2C_6H_4Cl$]$^+$, 100), 298 (5), 192 (87), 125 (29), 106 (46); HRMS (ESI-pos), *m/z* calcd. for $C_{24}H_{20}N_2O_5Cl$ [M]$^+$: 451.1055, measured: 451.1070.

1-(2-Fluorobenzyl)-4-(6,7-dimethoxy-2-oxo-2*H*-chromen-3-ylcarbamoyl)pyridinium bromide (9e). Prepared from **9** and 2-fluorobenzyl bromide, **9e** was obtained as yellow solid (32%). ^{1}H-NMR (DMSO-d_6, 400 MHz), δ: 10.68 (s, 1H), 9.35 (d, J = 4.0 Hz, 2H), 8.63 (s, 1H), 8.52 (d, J = 4.0 Hz, 2H), 7.69–7.64 (m, 1H), 7.58–7.52 (m, 1H), 7.39 (s, 1H), 7.38–7.32 (m, 2H), 7.31 (s, 1H), 6.07 (s,2H), 3.87 (s, 3H), 3.82 (s, 3H); ^{13}C-NMR (DMSO-d_6, 100 MHz), δ: 162.5, 160.5 (d, $^1J_{F,C}$ = 247.0 Hz), 157.6, 151.8, 148.8, 146.4, 146.3, 145.8(2C), 132.2 (d, $^3J_{F,CH}$ = 8.0 Hz), 131.5 (d, $^4J_{F,CH}$ = 3.0 Hz), 130.0, 126.8(2C), 125.3 (d, $^4J_{F,CH}$ = 3.0 Hz), 121.1 (d, $^2J_{F,C}$ = 15.0 Hz), 120.8, 116.0 (d, $^2J_{F,CH}$ = 21.0 Hz), 110.0, 109.1, 99.9, 58.2, 56.2, 56.0; FT-IR, ν_{max} (cm^{-1}): 3351, 1709, 1674, 1544, 1387, 1247, 997, 851, 755; MS (EI), *m/z* (relative intensity): 326 ([M-$CH_2C_6H_4F$]$^+$, 45), 192 (32), 109 (100), 97 (12), 83 (15); HRMS (ESI-pos), *m/z* calcd. for $C_{24}H_{20}N_2O_5F$ [M]$^+$: 435.1351, measured: 435.1361.

1-(3-Fluorobenzyl)-4-(6,7-dimethoxy-2-oxo-2*H*-chromen-3-ylcarbamoyl)pyridinium bromide (9f). Prepared from **9** and 3-fluorobenzyl bromide, **9f** was obtained as yellow solid (30%). ^{1}H-NMR (DMSO-d_6, 400 MHz), δ: 10.66 (s, 1H), 9.45 (d, J = 8.0 Hz, 2H), 8.62 (s, 1H), 8.53 (d, J = 8.0 Hz, 2H), 7.56–7.51 (m, 2H), 7.45–7.43 (m, 1H), 7.40 (s, 1H), 7.33–7.29 (m, 1H), 7.13 (s, 1H), 5.99 (s, 2H), 3.87 (s, 3H), 3.82 (s, 3H); ^{13}C-NMR (DMSO-d_6, 100 MHz), δ: 162.5, 162.2 (d, $^{1}J_{F,C}$ = 244.0 Hz), 157.6, 151.8, 148.6, 146.5, 146.3, 145.7(2C), 136.5 (d, $^{3}J_{F,C}$ = 8.0 Hz), 131.4 (d, $^{3}J_{F,CH}$ = 8.0 Hz), 130.0, 126.8(2C), 125.1 (d, $^{4}J_{F,CH}$ = 3.0 Hz), 120.8, 116.4 (d, $^{2}J_{F,CH}$ = 21.0 Hz), 115.9, 111.0, 109.1, 99.9, 62.6, 56.2, 56.0; FT-IR, ν_{max} (cm^{-1}): 3335, 1713, 1676, 1541, 1454, 1276, 1250; MS (EI), *m/z* (relative intensity): 326 ([M-$CH_2C_6H_4F$]$^+$, 100), 192 (42), 109 (73), 88 (27), 71 (19); HRMS (ESI-pos), *m/z* calcd. for $C_{24}H_{20}N_2O_5F$ [M]$^+$: 435.1351, measured: 435.1345.

1-(4-Fluorobenzyl)-4-(6,7-dimethoxy-2-oxo-2*H*-chromen-3-ylcarbamoyl)pyridinium bromide (9g). Prepared from **9** and 4-fluorobenzyl bromide, **9g** was obtained as yellow solid (37%). ^{1}H-NMR (DMSO-d_6, 300 MHz), δ: 10.62 (s, 1H), 9.38 (d, J = 6.6 Hz, 2H), 8.62 (s, 1H), 8.50 (d, J = 6.6 Hz, 2H), 7.68 (dd, J = 8.7, 5.4 Hz, 2H), 7.39 (s, 1H), 7.33 (t, J = 9.0 Hz, 2H), 7.13 (s, 1H), 5.93 (s, 2H), 3.87 (s, 3H), 3.82 (s, 3H); ^{13}C-NMR (DMSO-d_6, 75 MHz), δ: 162.6 (d, $^{1}J_{F,C}$ = 245.0 Hz), 162.5, 157.6, 151.9, 148.6, 146.5, 146.3, 145.5(2C), 131.5 (d, $^{3}J_{F,CH}$ = 8.3 Hz, 2C), 130.4 (d, $^{4}J_{F,C}$ = 3.0 Hz), 130.0, 126.8(2C), 120.8, 116.2 (d, $^{2}J_{F,CH}$ = 21.0 Hz, 2C), 111.0, 109.1, 99.9, 62.7, 56.2, 56.0; FT-IR, ν_{max} (cm^{-1}): 3340, 1710, 1675, 1540, 1450, 1270, 987; MS (EI), *m/z* (relative intensity): 326 ([M-$CH_2C_6H_4F$]$^+$, 35),

192 (45), 109 (100), 88 (10), 71 (15); HRMS (ESI-pos), *m/z* calcd. for $C_{24}H_{20}N_2O_5F$ $[M]^+$: 435.1351, measured: 435.1349.

1-(2,3-Difluorobenzyl)-4-(6,7-dimethoxy-2-oxo-2*H*-chromen-3-ylcarbamoyl)pyridinium bromide (9h). Prepared from **9** and 2,3-difluorobenzyl bromide, **9h** was obtained as yellow solid (25%). ^{1}H-NMR (DMSO-d_6, 400 MHz), δ: 10.67 (s, 1H), 9.36 (d, J = 4.0 Hz, 2H), 8.63 (s, 1H), 8.53 (d, J = 4.0 Hz, 2H), 7.62–7.56 (m, 1H), 7.49–7.42 (m, 1H), 7.40 (s, 1H), 7.38–7.33 (m, 1H), 7.13 (s, 1H), 6.13 (s, 2H), 3.87 (s, 3H), 3.82 (s, 3H); ^{13}C-NMR (DMSO-d_6, 100 MHz), δ: 162.4, 157.6, 151.8, 149.7 (dd, $^1J_{F,C}$ = 245.0 Hz, $^2J_{F,C}$ = 12.0 Hz), 148.9, 148.4 (dd, $^1J_{F,C}$ = 248.0 Hz, $^2J_{F,C}$ = 13.0 Hz), 146.5, 146.3, 146.0(2C), 130.1, 126.8(2C), 126.5 (dd, $^3J_{F,CH}$ = 4.0 Hz, $^4J_{F,CH}$ = 2.0 Hz), 125.8 (dd, $^3J_{F,CH}$ = 7.0 Hz, $^4J_{F,CH}$ = 4.0 Hz), 123.4 (d, $^2J_{F,C}$ = 11.0 Hz), 120.8, 119.2 (d, $^2J_{F,CH}$ = 16.0 Hz), 111.0, 109.1, 99.9, 57.5, 56.2, 56.0; FT-IR, ν_{max} (cm^{-1}): 3347, 1710, 1674, 1542, 1386, 1247, 995; MS (EI), *m/z* (relative intensity): 326 ($[M\text{-}CH_2C_6H_3F_2]^+$, 41), 192 (22), 127 (100), 97 (30), 73 (25), 57 (22); HRMS (ESI-pos), *m/z* calcd. for $C_{24}H_{19}N_2O_5F_2$ $[M]^+$: 453.1257, measured: 453.1261.

1-(2,6-Difluorobenzyl)-4-(6,7-dimethoxy-2-oxo-2*H*-chromen-3-ylcarbamoyl)pyridinium bromide (9i). Prepared from **9** and 2,6-difluorobenzyl bromide, **9i** was obtained as yellow solid (25%). ^{1}H-NMR (DMSO-d_6, 300 MHz), δ: 10.71 (s, 1H), 9.28 (d, J = 6.3 Hz, 2H), 8.62 (s, 1H), 8.49 (d, J = 6.3 Hz, 2H), 7.70–7.60 (m, 1H), 7.39 (s, 1H), 7.33–7.27 (m, 2H), 7.13 (s, 1H), 6.11 (s, 2H), 3.82 (s, 3H), 3.82 (s, 3H); ^{13}C-NMR (DMSO-d_6, 75 MHz), δ: 162.5, 160.9 (d, $^1J_{F,C}$ = 248.9 Hz), 160.8 (d, $^1J_{F,C}$ = 248.9 Hz), 157.6, 151.8, 148.9, 146.4, 146.3, 145.9(2C), 133.3

(t, $^3J_{F,CH}$ = 10.5 Hz), 130.1, 126.8(2C), 120.8, 112.4 (d, $^2J_{F,CH}$ = 24.2 Hz), 111.0, 109.6 (t, $^2J_{F,C}$ = 18.6 Hz), 109.1, 99.9, 56.2, 56.0, 52.3; FT-IR, v_{max} (cm^{-1}): 3345, 1695, 1668 1541, 1246, 998, 840; MS (EI), *m/z* (relative intensity): 326 ([M-$CH_2C_6H_3F_2$]$^+$, 93), 298 (4), 192 (90), 127 (100), 106 (51); HRMS (ESI-pos), *m/z* calcd. for $C_{24}H_{19}N_2O_5F_2$ [M]$^+$: 453.1257, measured: 453.1255.

AChE inhibition assay

AChE (*Electrophorus electricus*, Type VI-S, lyophilized powder) was purchased from Sigma-Aldrich, USA. Acetylthiocholine, 5,5′-dithiobis(2-nitrobenzoic acid), sodium dihydrogen phosphate, disodium hydrogen phosphate, and sodium hydrogen carbonate were obtained from Fluka, Switzerland. AChE inhibitory activity was measured with a Molecular Devices Spectra Max Plus 384 spectrophotometer (CA, USA) based on Ellman's method with slight modifications. For each assay, 200 µL of the assay medium containing 100 µL of 0.1 M phosphate buffer (pH 8), 20 µL of 3.3 mM 5,5′-dithiobis(2-nitrobenzoic acid) in 0.1 M phosphate buffer (pH 7) containing 6 mM $NaHCO_3$, 20 µL of the test compounds at different concentrations in methanol, and 40 µL of 0.15 unit/mL AChE in phosphate buffer (pH 8) were incubated at 37 °C for 20 min. Subsequently, 20 µL of 5 mM acetylthiocholine iodide in water was added, and the absorbance was measured at 412 nm every 20 s for 3 min. The rate of each reaction was calculated by Microplate Manager software. The blank percentage of inhibition of the tested compounds was determined by comparing the rate of the sample to the blank. The IC_{50} value was determined by nonlinear regression of the log inhibitor concentration versus the percentage of inhibition using GraphPad Prism6. Donepezil hydrochloride and tacrine were applied as the reference compounds. All samples were assayed in triplicate.

Molecular docking studies

The compounds were constructed in the DS Visualizer program and these structures were optimized by the B3LYP/6-31G** method using the Gaussian 09 program. To prepare the rhAChE structure, we retrieved the crystal structure of the rhAChE in complex with donepezil (PDB code 4ey7) from the Protein Data Bank. From the crystal structure, the binding of donepezil indicated the presence of a binding site in rhAChE. Molecular docking with the GOLD v5.2.2 program was applied to investigate the orientation of the compounds in the rhAChE binding site. This binding site is defined by the center of mass of donepezil; this expanded from the center with a radius of 10 Å. The default parameters of the automatic settings and a genetic algorithm (GA) run of 15 were used for setting the GA parameters. The GoldScore fitness function was used to determine the fitness score of the docked compound. The parameters were validated by docking donepezil back into the binding site and evaluating their effects as RMSD. Subsequently, these GA parameters were applied to dock compounds into the rhAChE binding site. The conformation with the highest GoldScore was selected for analyzing the interaction between surrounding amino acids.

References

[1] Linch, F. W. *J. Chem. Soc., Trans.* **1912,** *101,* 1758–1765.

[2] Kadhum, A.A.H.; Mohamad, A. B.; Al-Amiery, A. A.; Takriff, M. S. *Molecules* **2011**, *16*, 6969–6984.

[3] Jackson, Y. A. *Heterocycles* **1995**, *41*, 1979–1986.

^{1}H and ^{13}C NMR spectra (DMSO-d_6, 300 MHz) of compound **4**

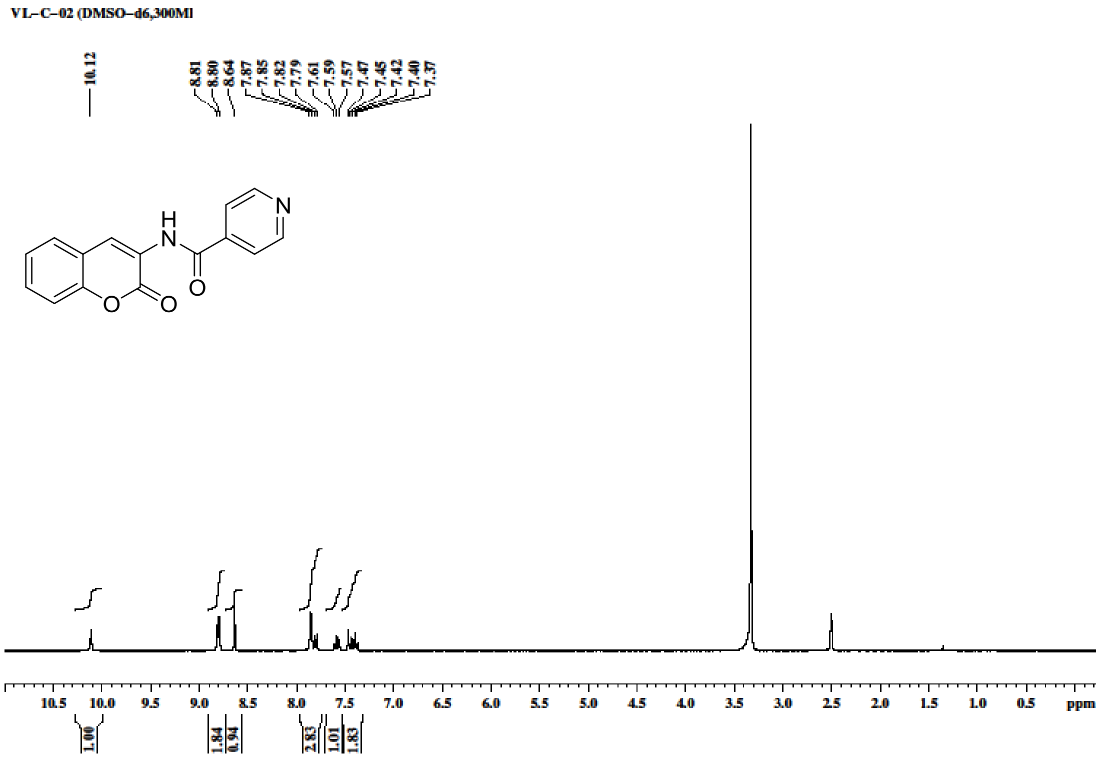

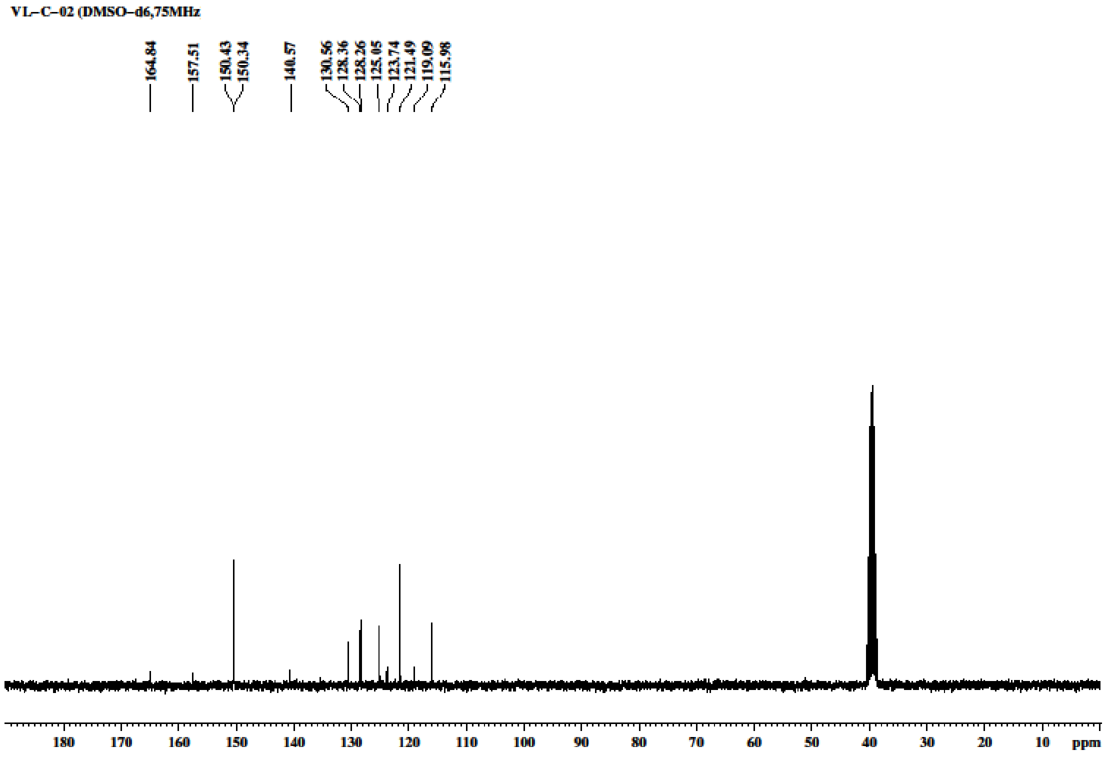

^{1}H and ^{13}C NMR (DMSO-d_6, 300 MHz) of compound **4a**

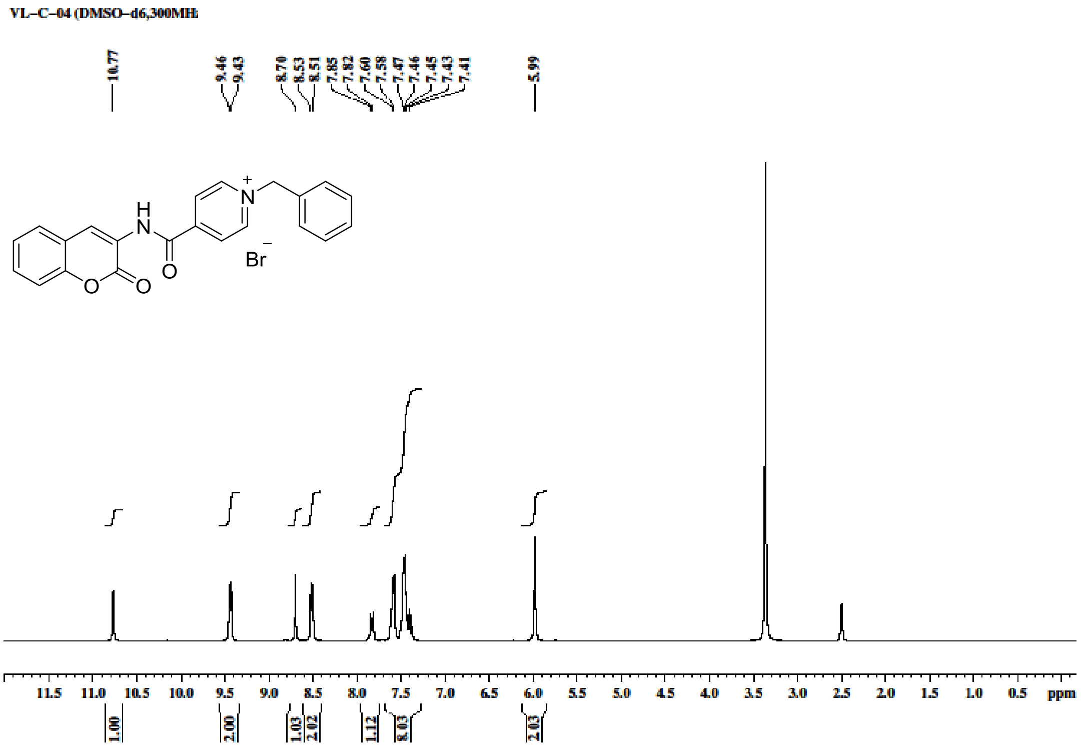

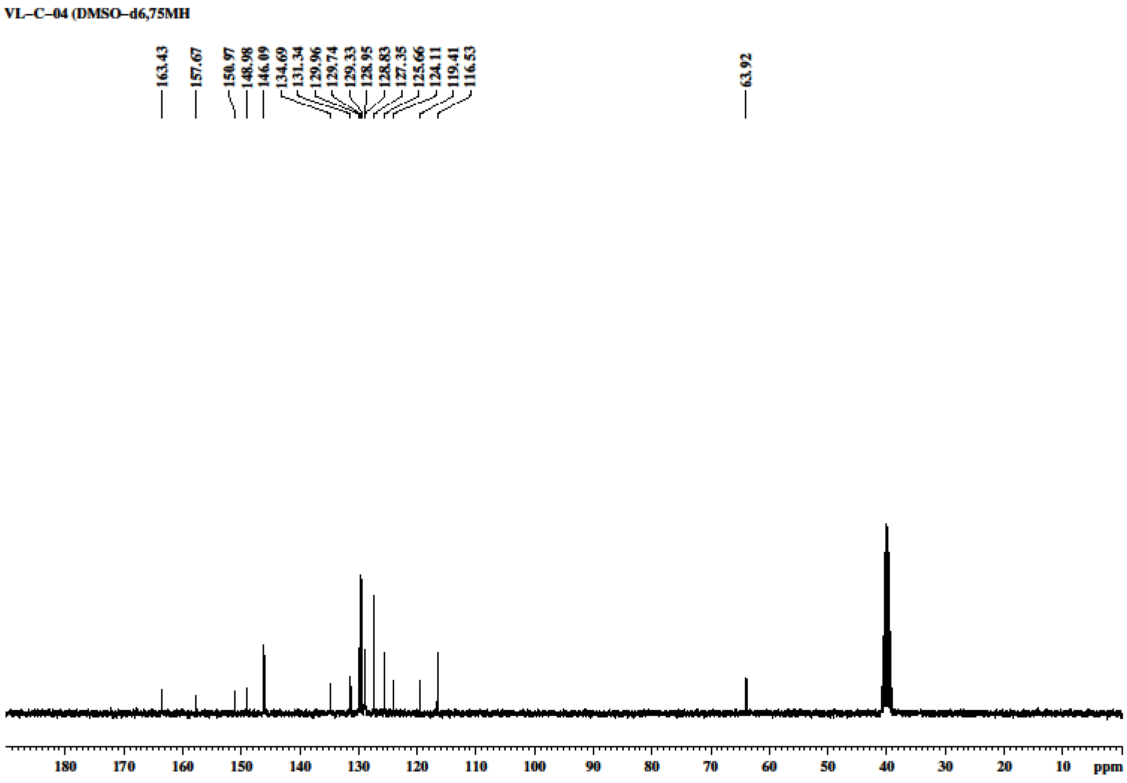

^{1}H and ^{13}C NMR spectra ($CDCl_3$, 300 MHz) of compound **5**

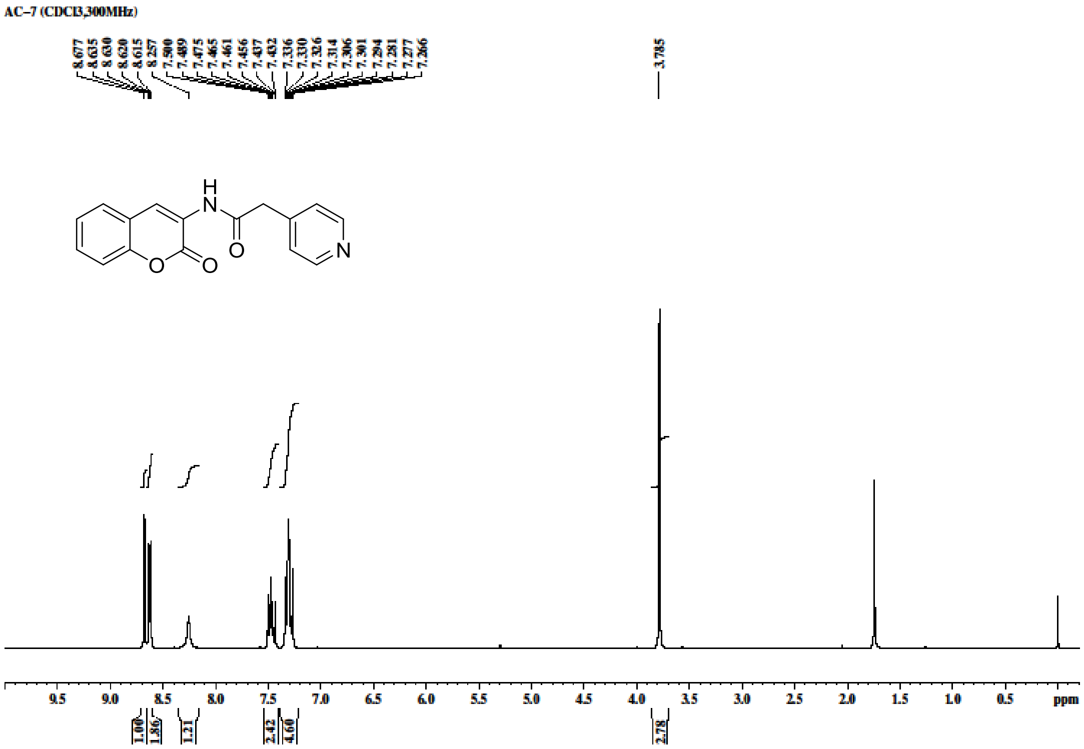

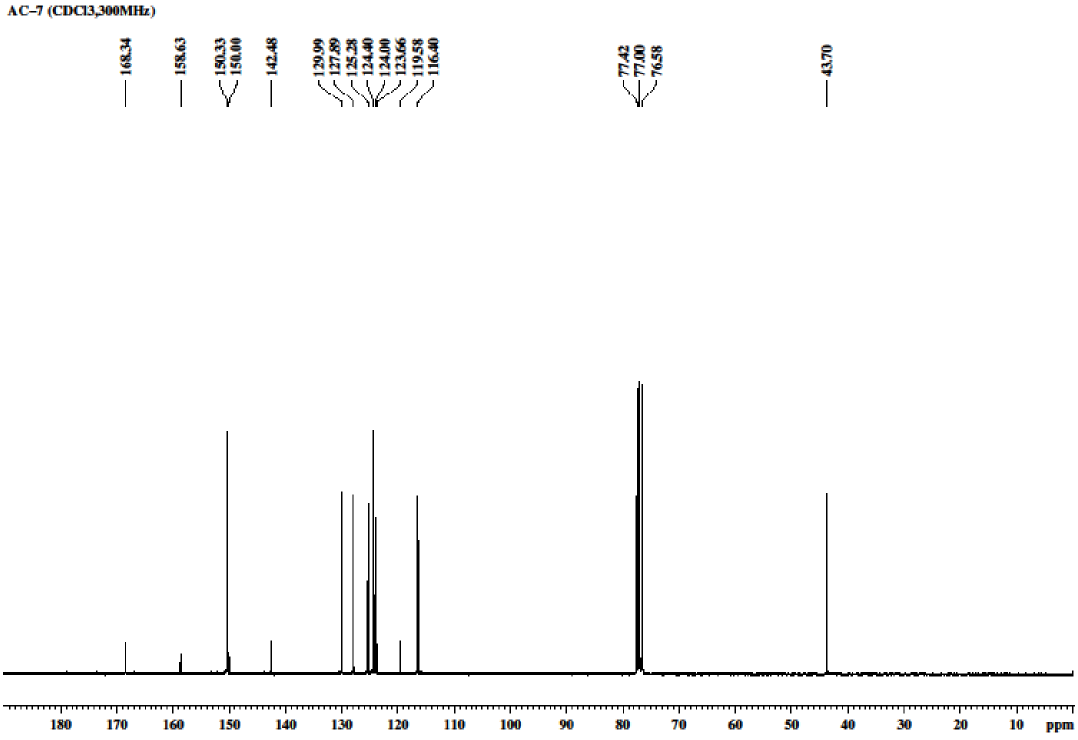

^{1}H and ^{13}C NMR (DMSO-d_6, 300 MHz) of compound **5a**

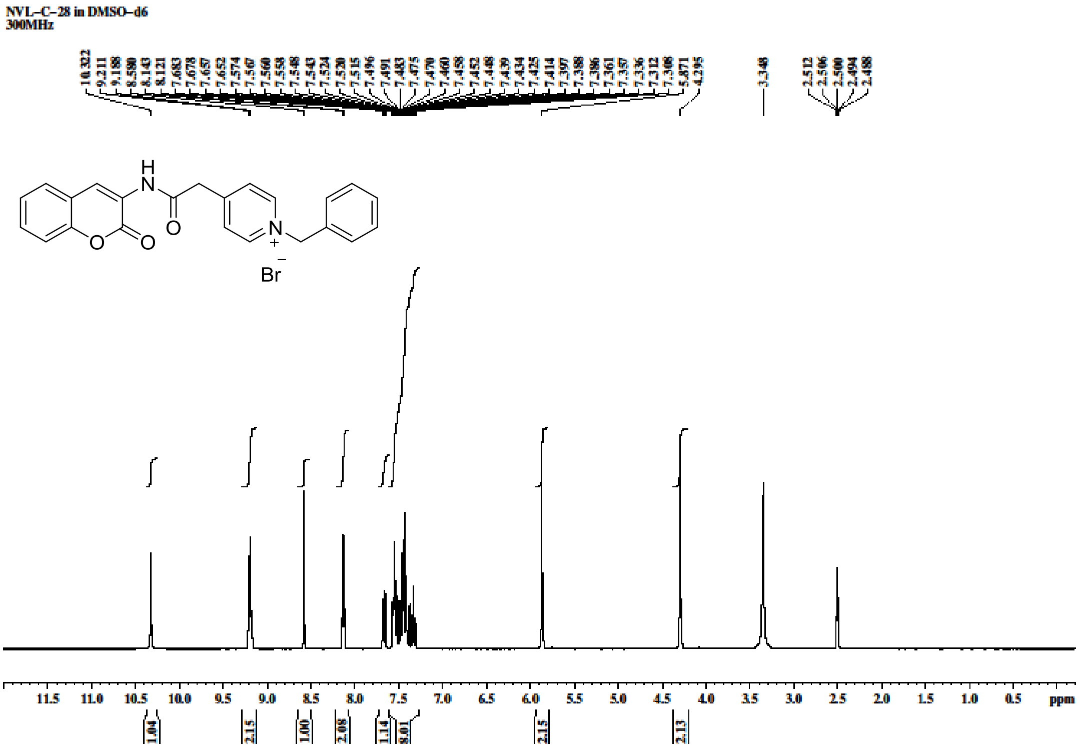

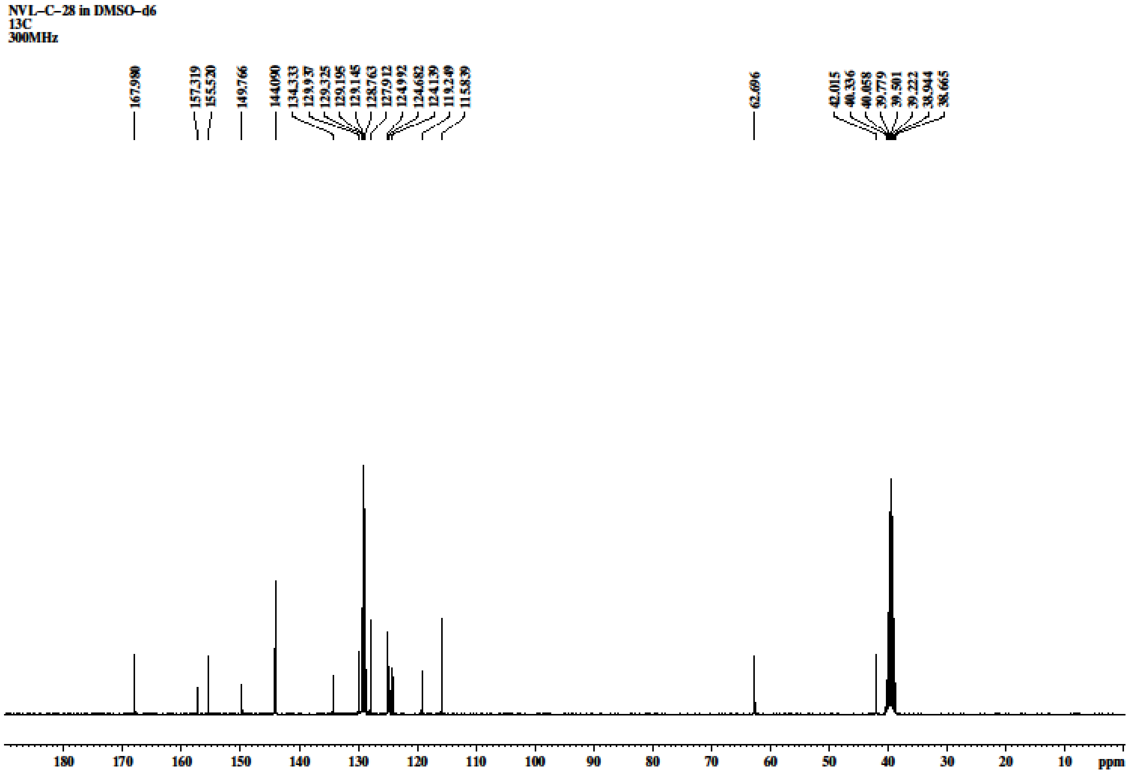

^{1}H and ^{13}C NMR spectra ($CDCl_3$, 300 MHz) of compound **7**

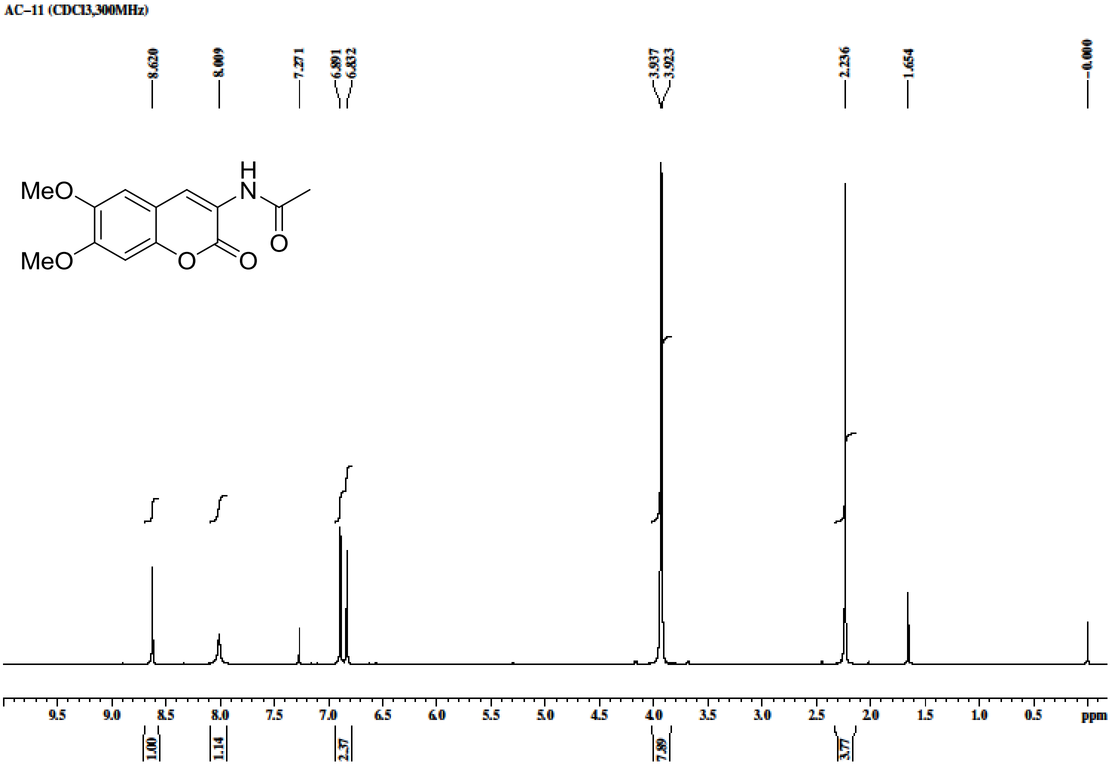

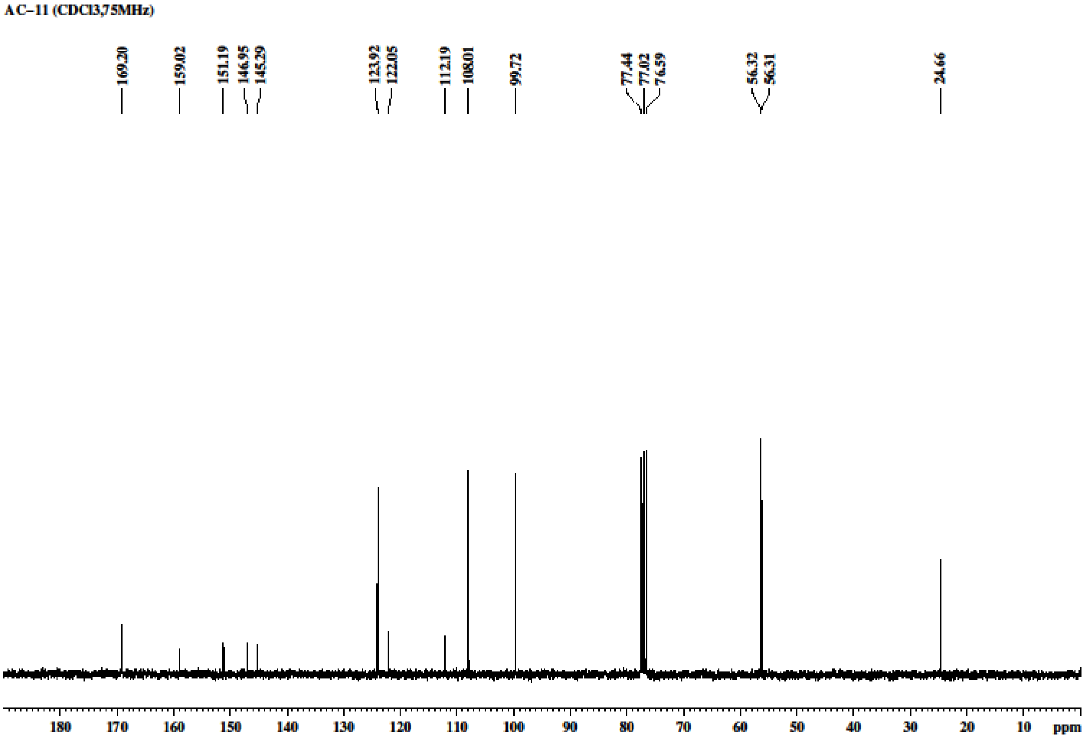

^{1}H and ^{13}C NMR spectra ($CDCl_3$, 300 MHz) of compound **8**

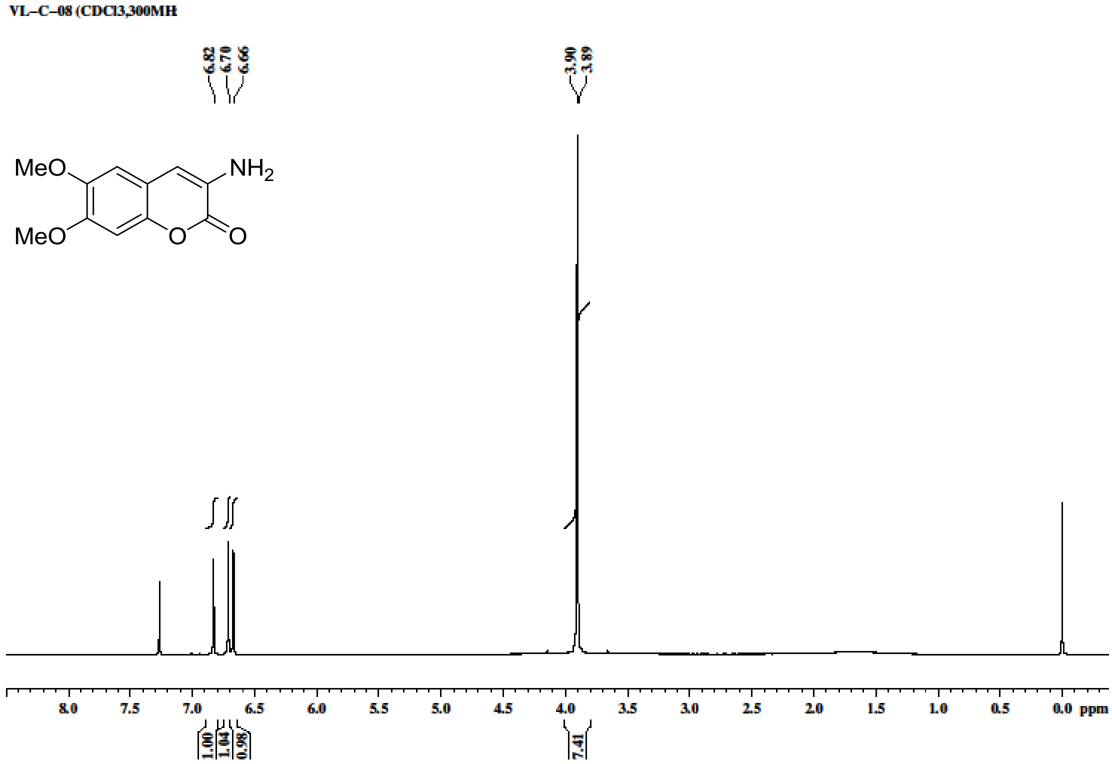

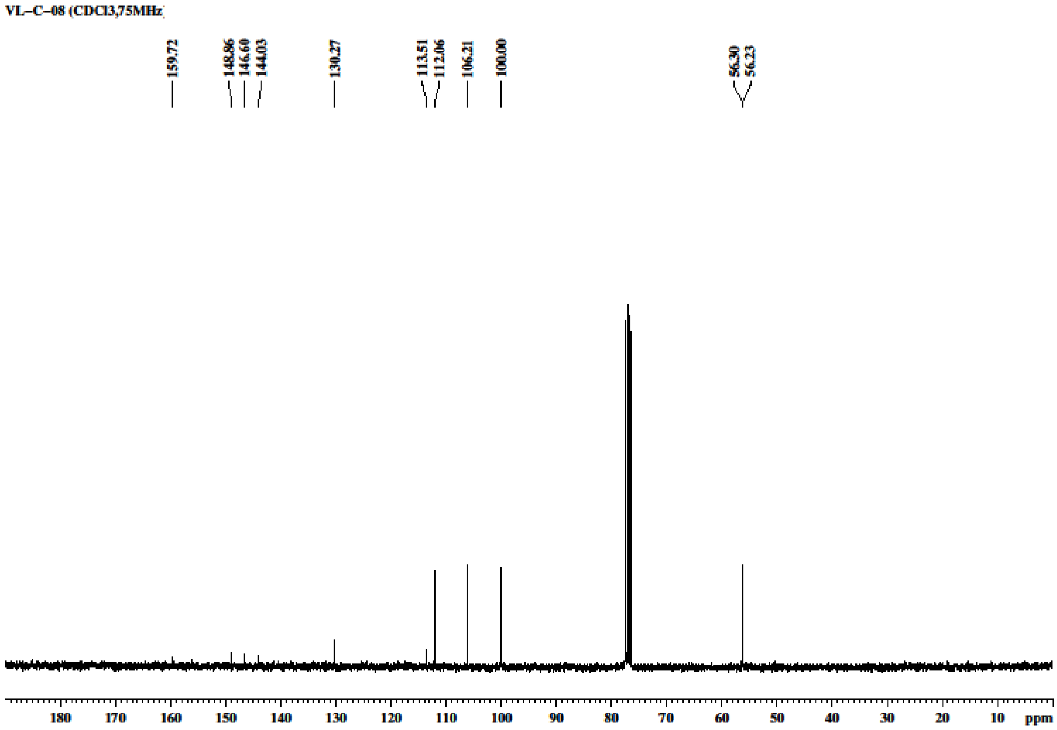

^{1}H and ^{13}C NMR (DMSO-d_6, 300 MHz) of compound **9**

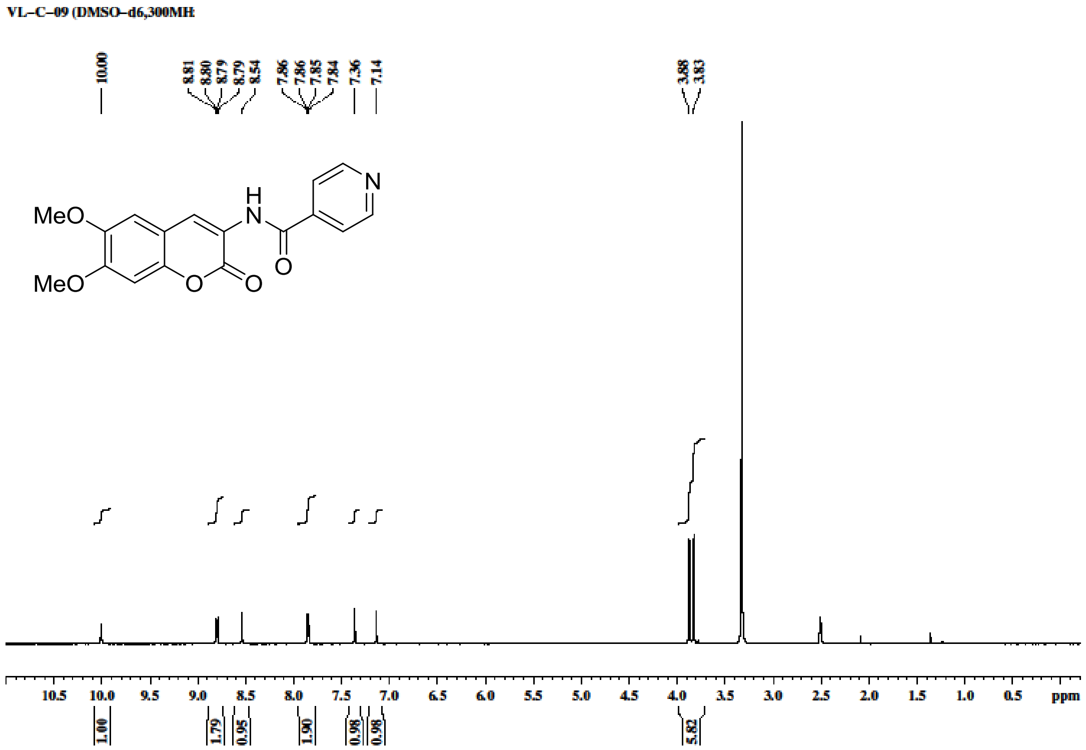

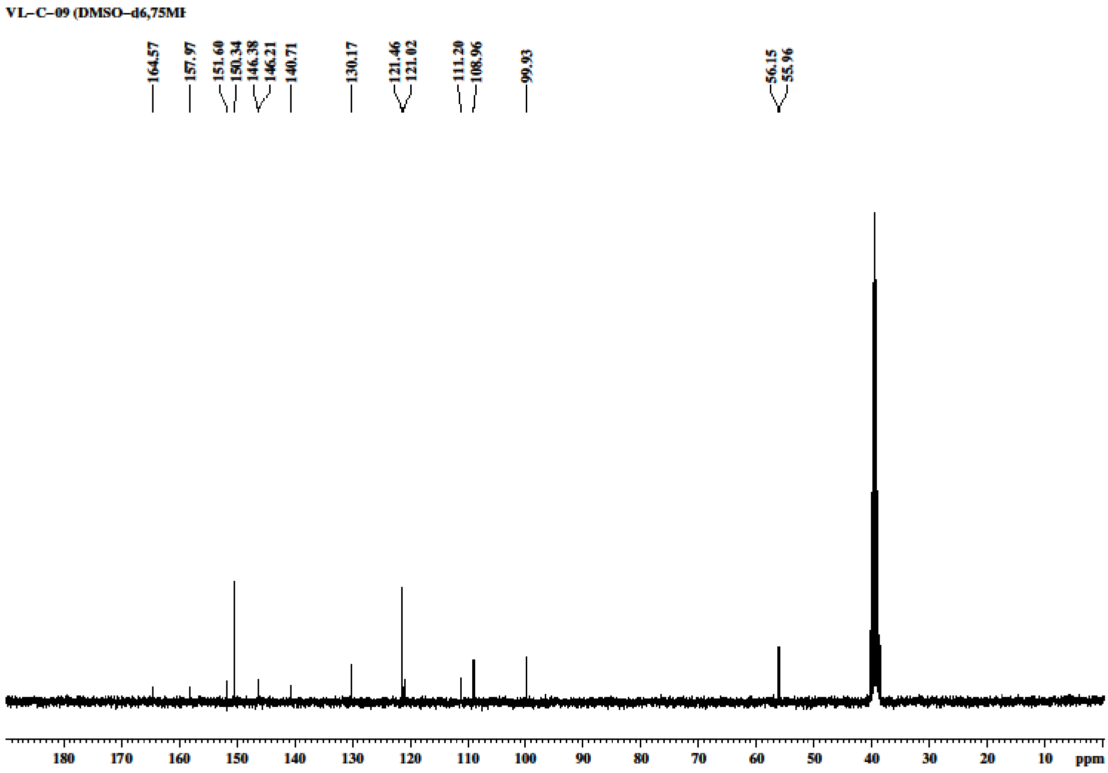

^{1}H and ^{13}C NMR (DMSO-d_6, 300 MHz) of compound **9a**

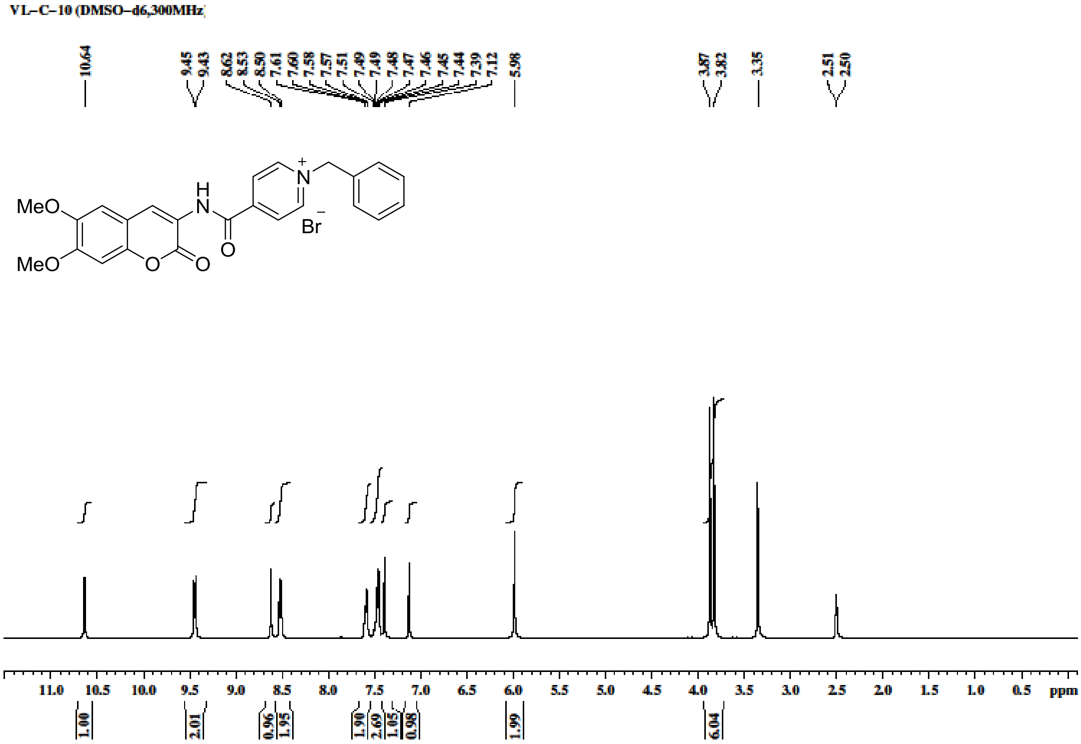

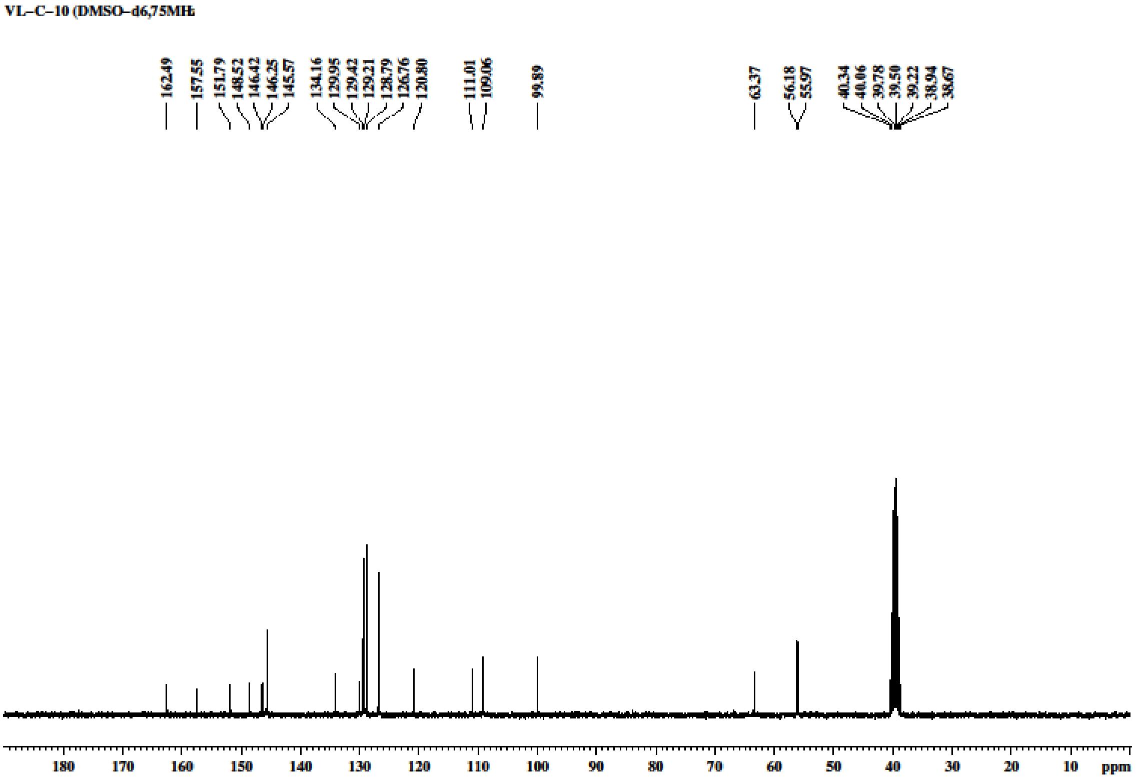

^{1}H and ^{13}C NMR (DMSO-d_6, 400 MHz) of compound **9b**

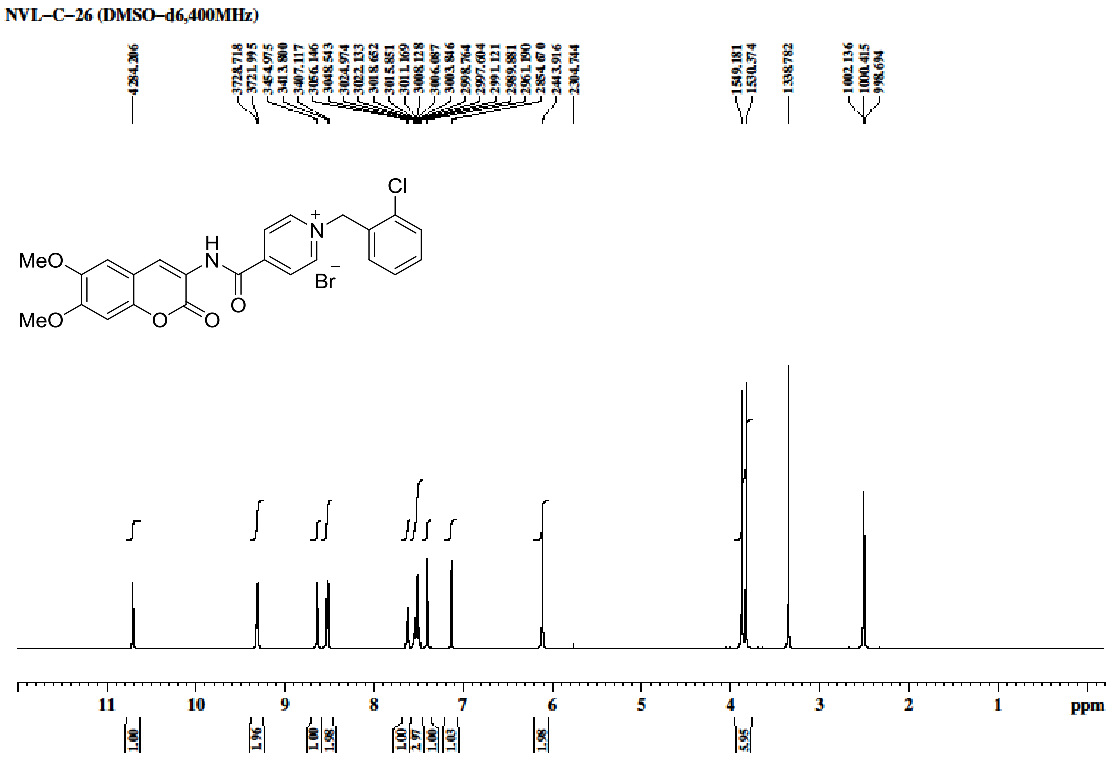

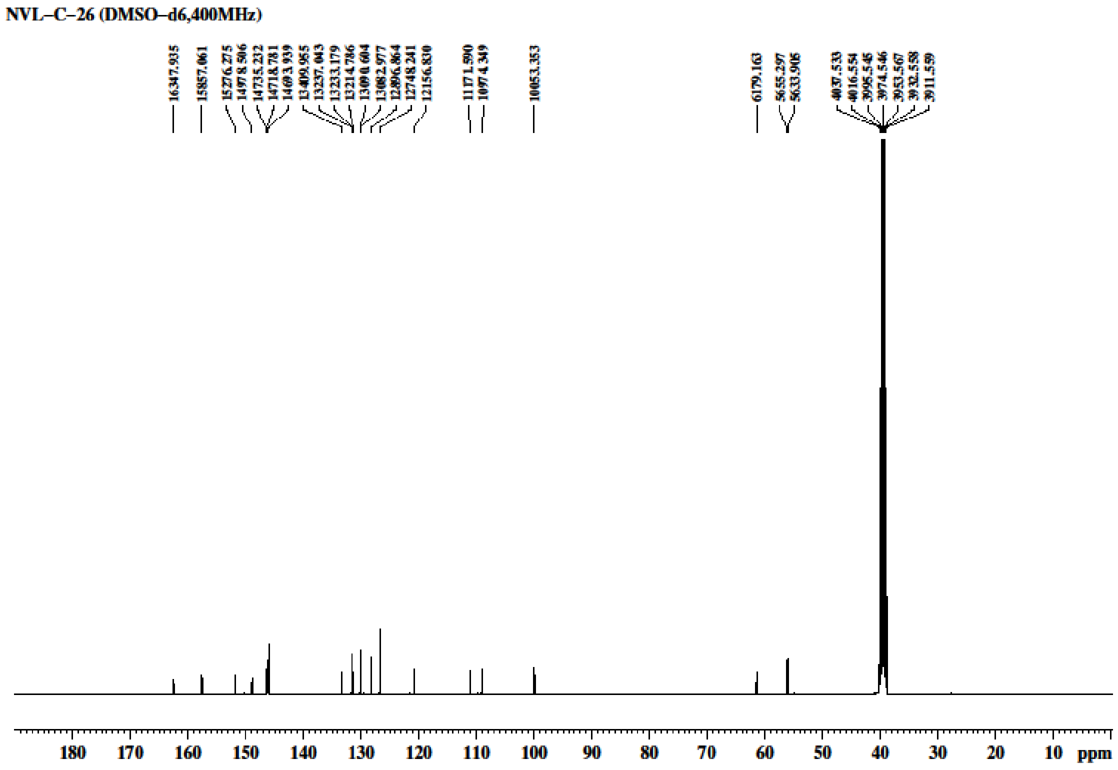

^{1}H and ^{13}C NMR (DMSO-d_6, 400 MHz) of compound **9c**

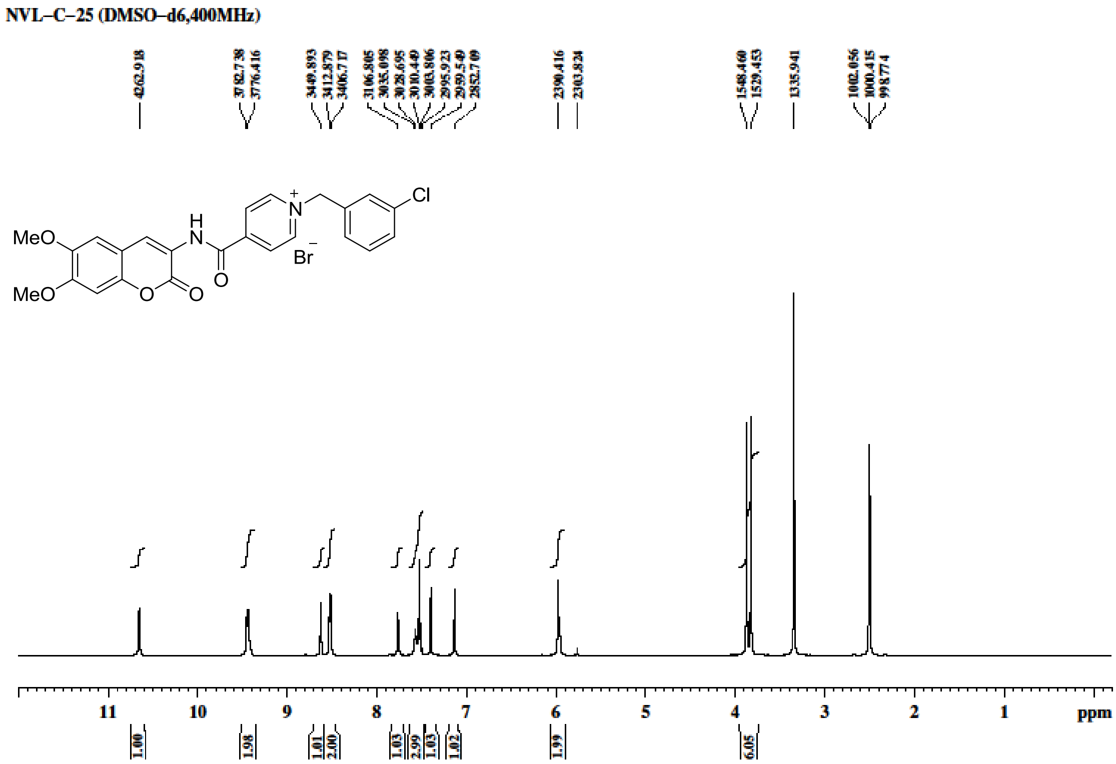

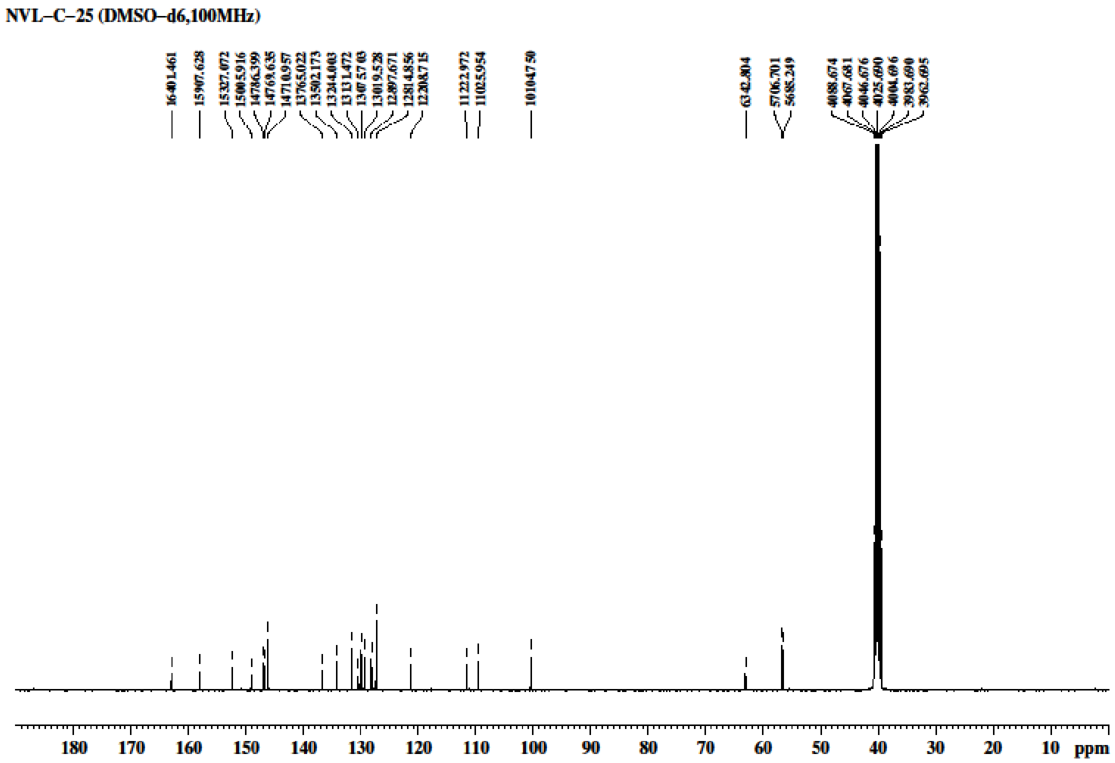

^{1}H and ^{13}C NMR (DMSO-d_6, 600 MHz) of compound **9d**

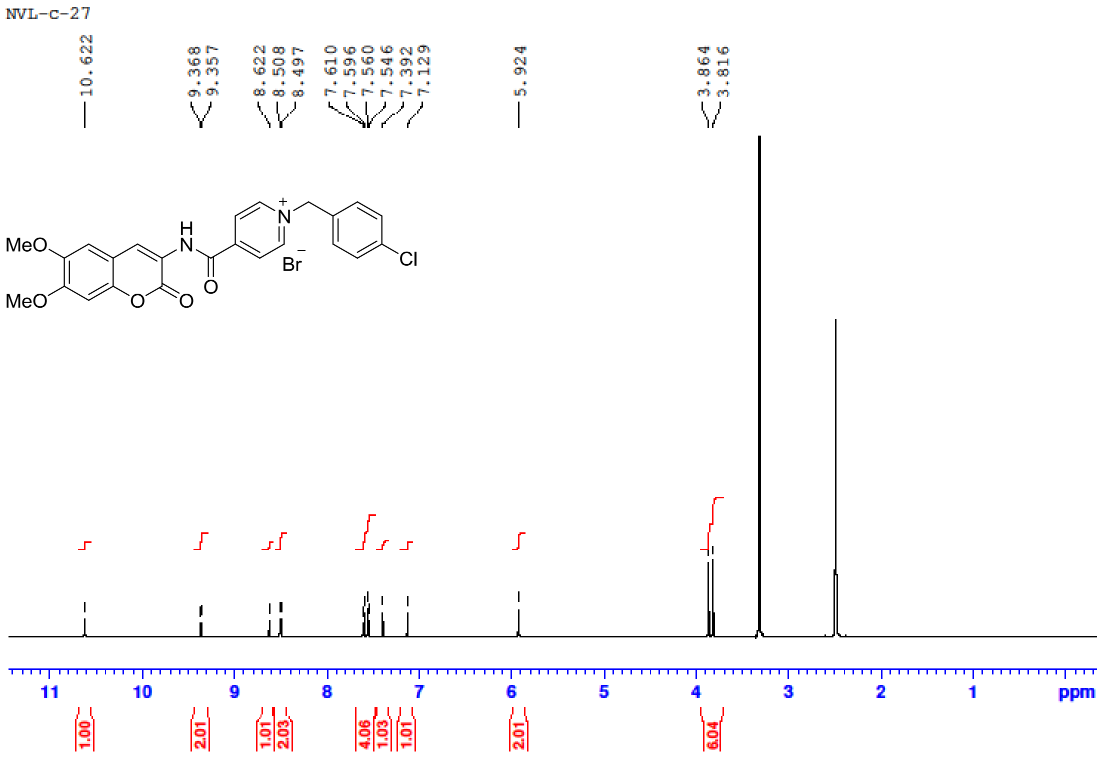

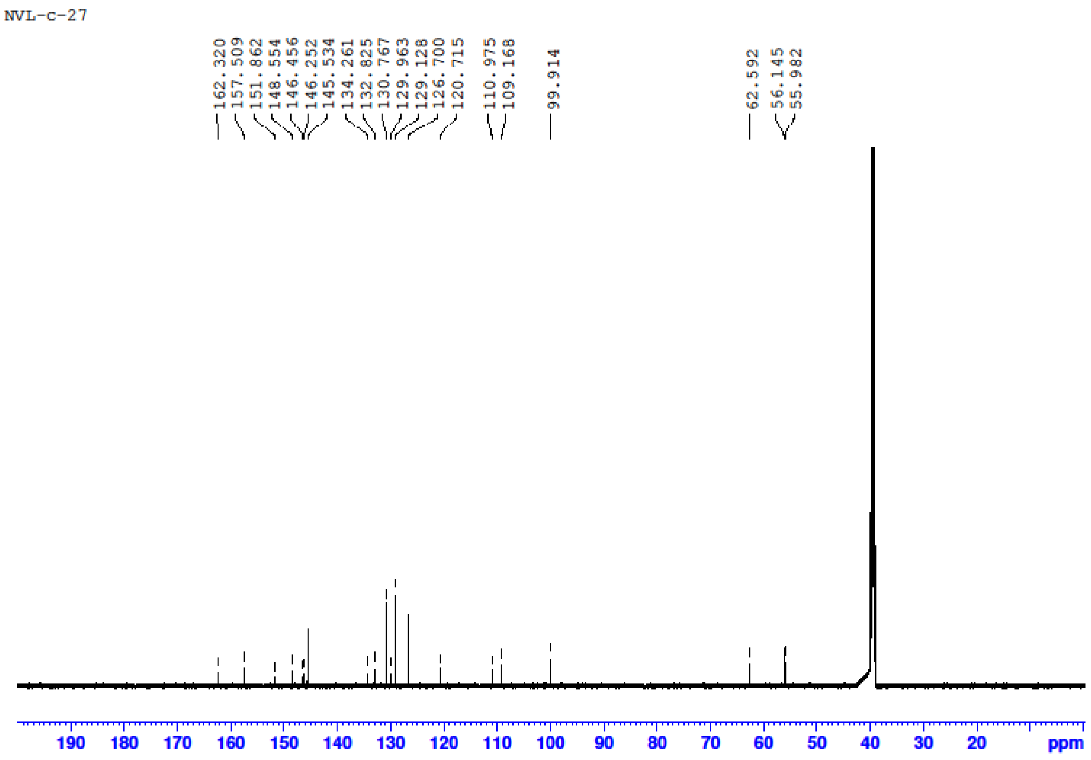

^{1}H and ^{13}C NMR (DMSO-d_6, 400 MHz) of compound **9e**

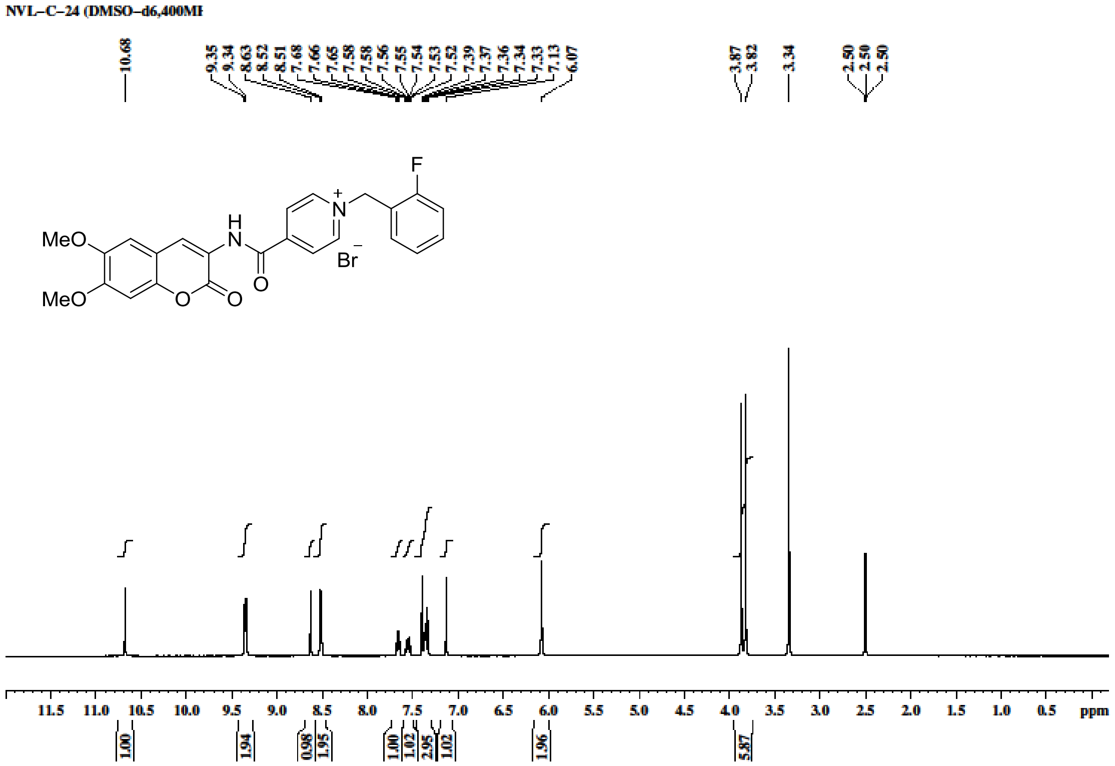

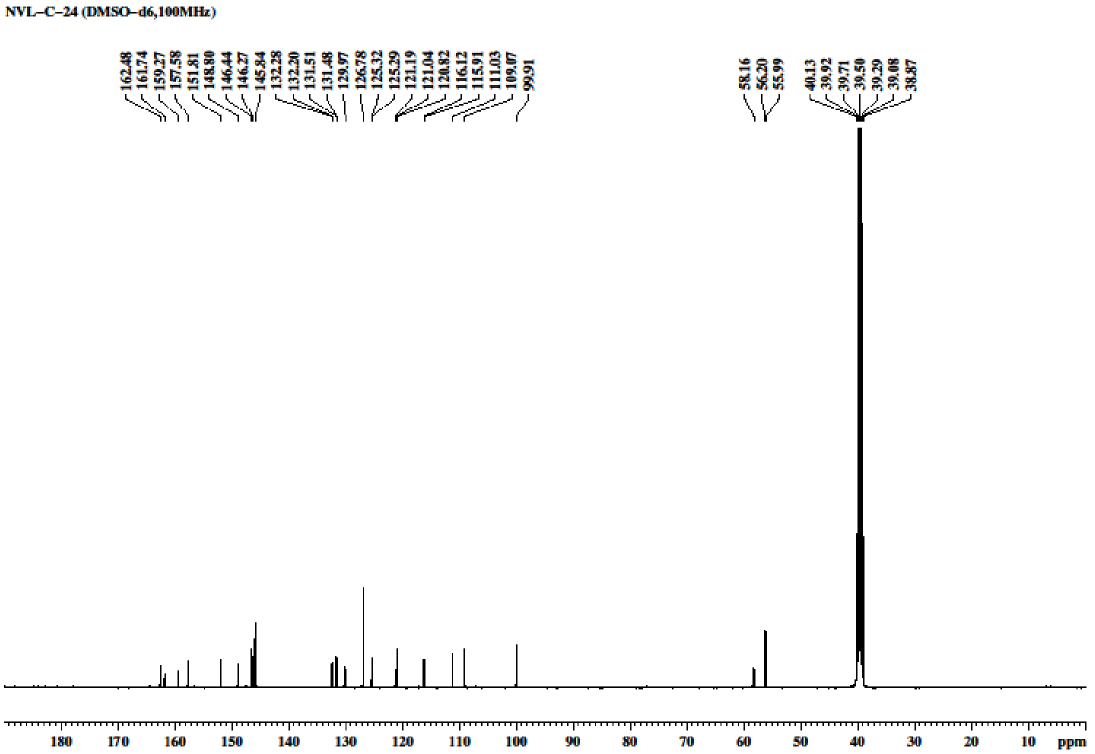

^{1}H and ^{13}C NMR (DMSO-d_6, 400 MHz) of compound **9f**

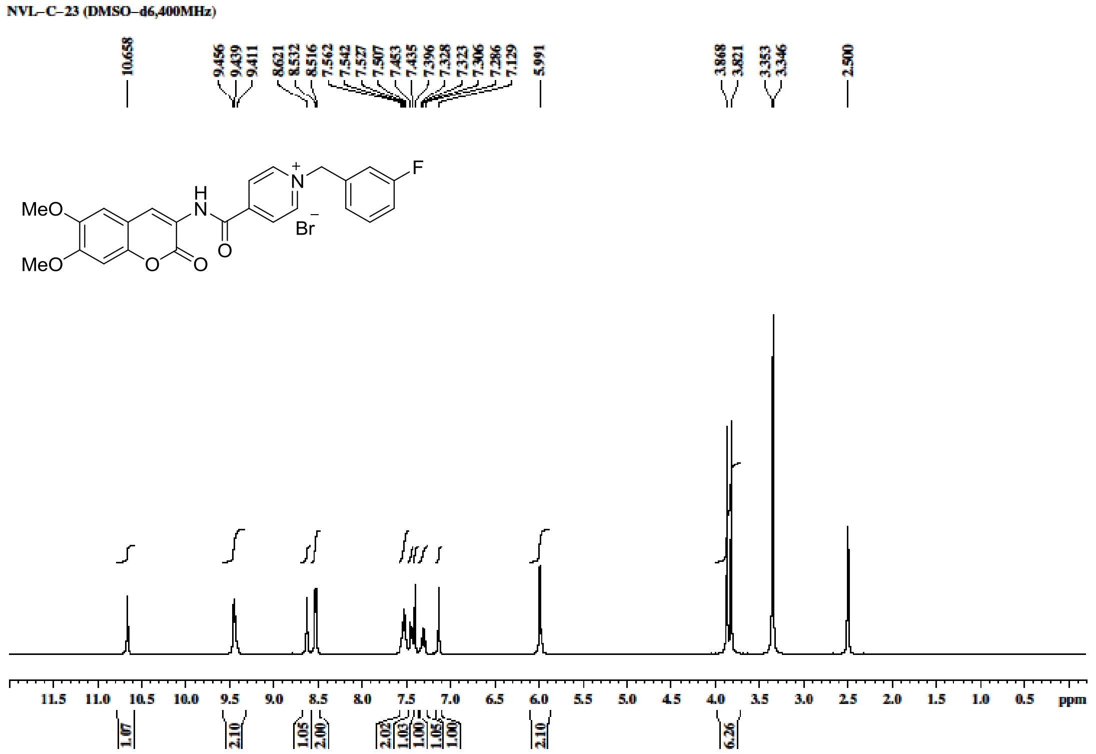

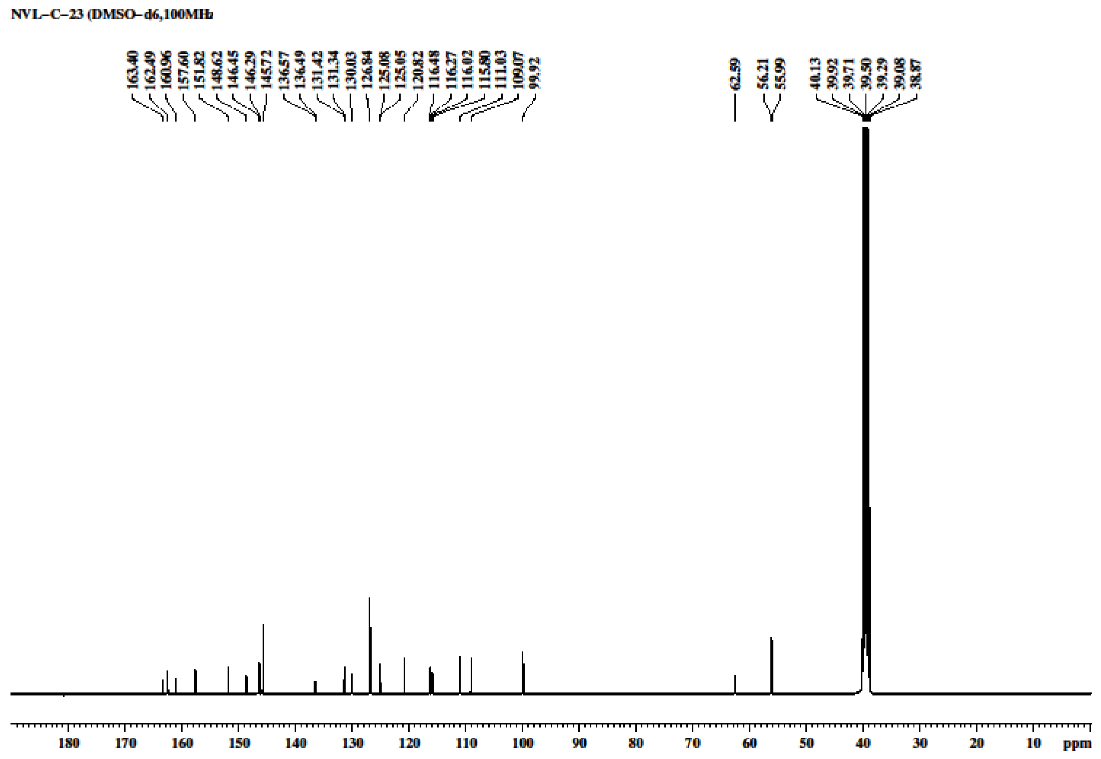

^{1}H and ^{13}C-NMR (DMSO-d_6, 300 MHz) of compound **9g**

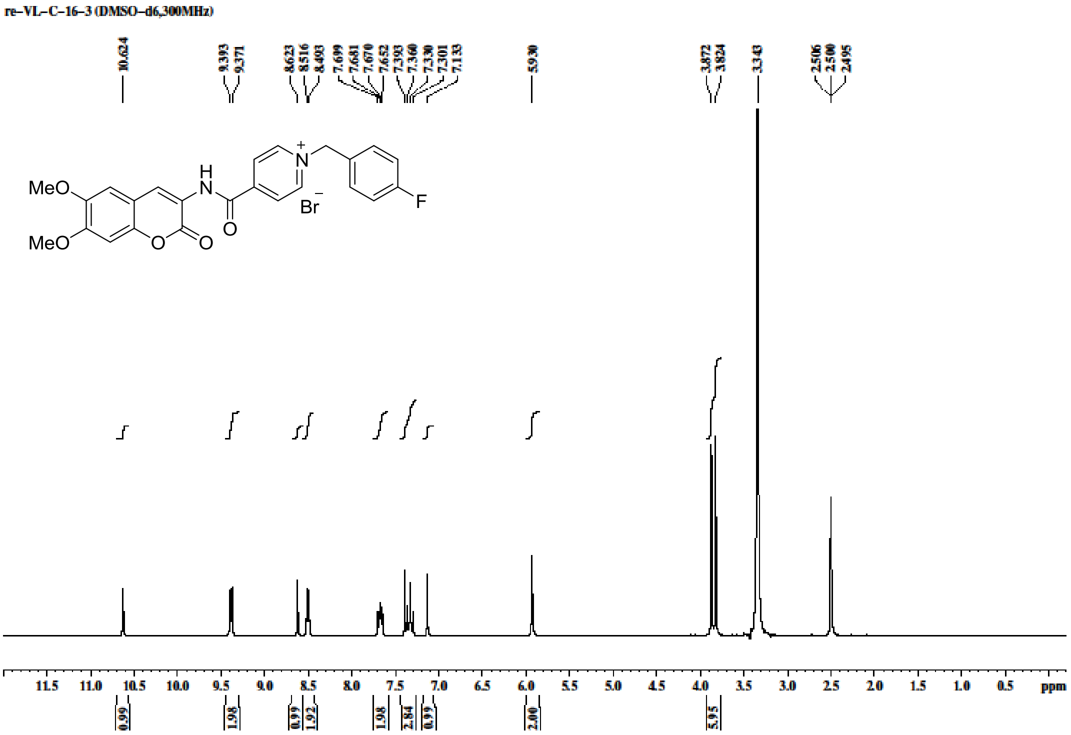

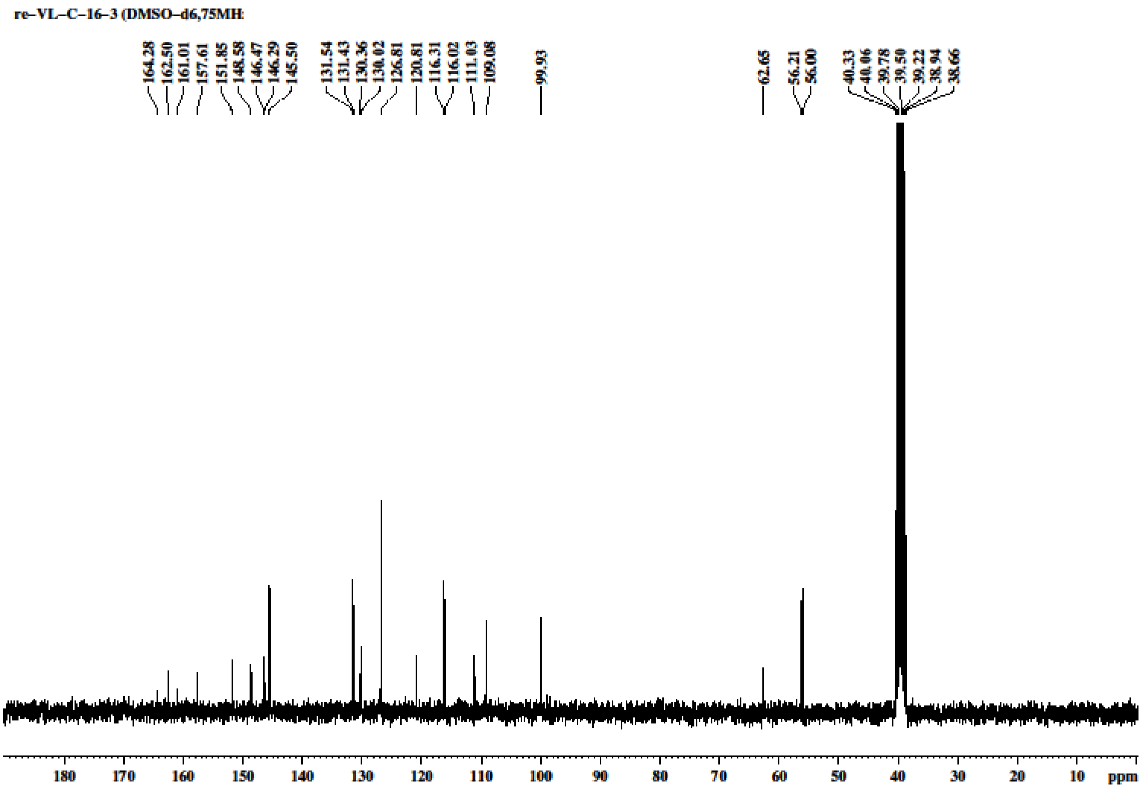

^{1}H and ^{13}C NMR (DMSO-d_6, 400MHz) of compound **9h**

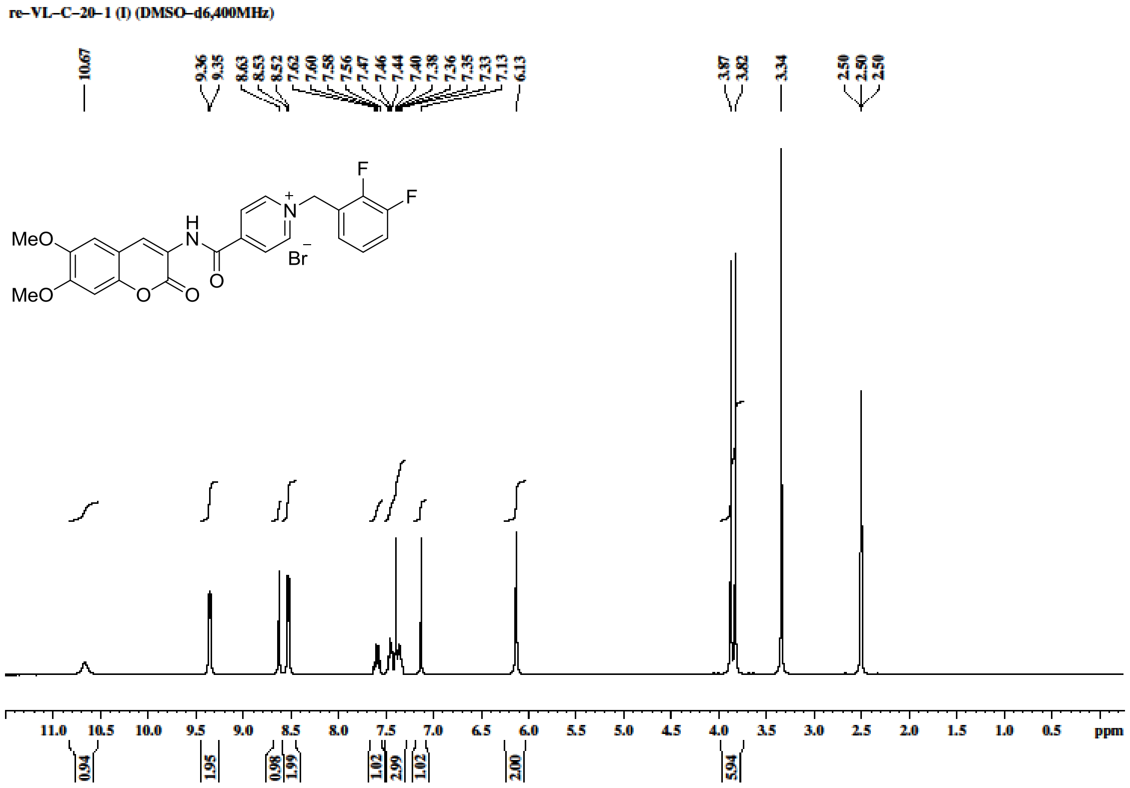

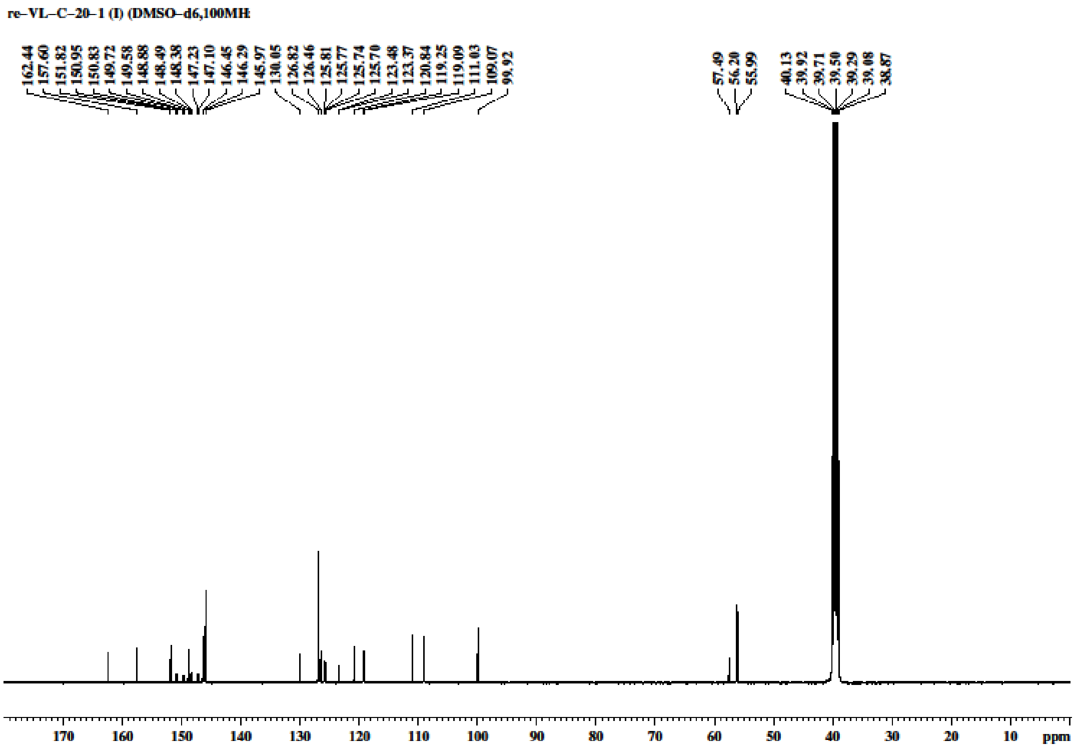

^{1}H and ^{13}C NMR (DMSO-d_6, 300 MHz) of compound **9i**

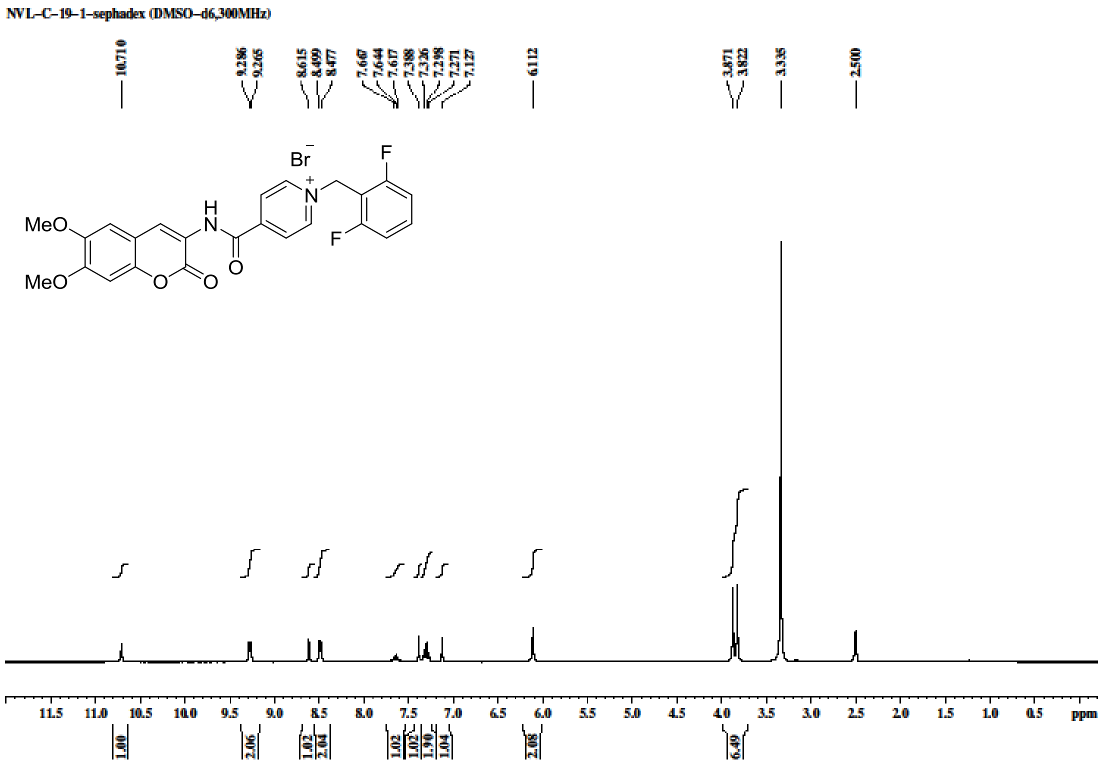

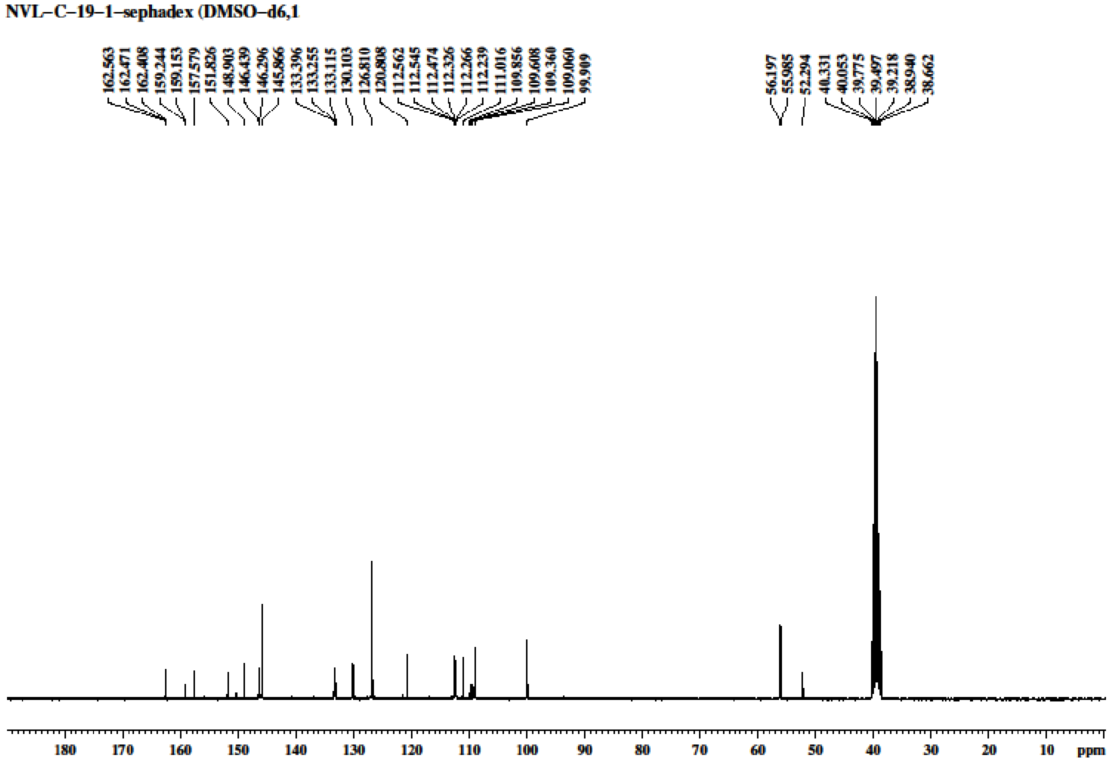

^{1}H and ^{13}C NMR (DMSO-d_6, 300 MHz) of compound **10**

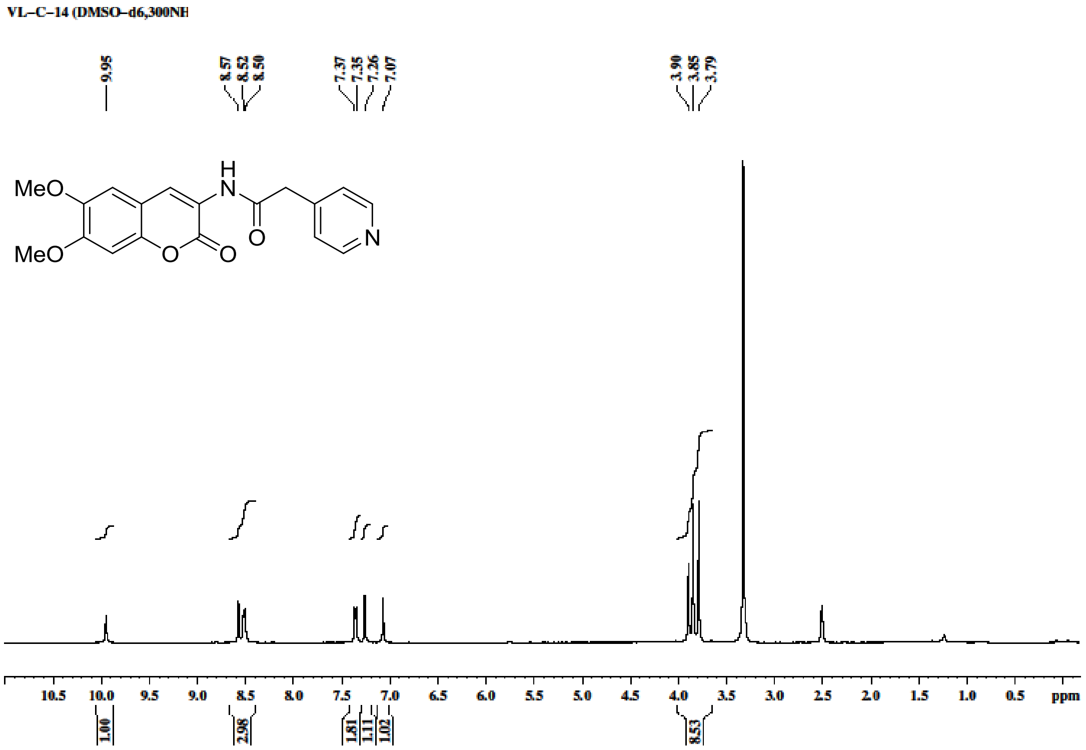

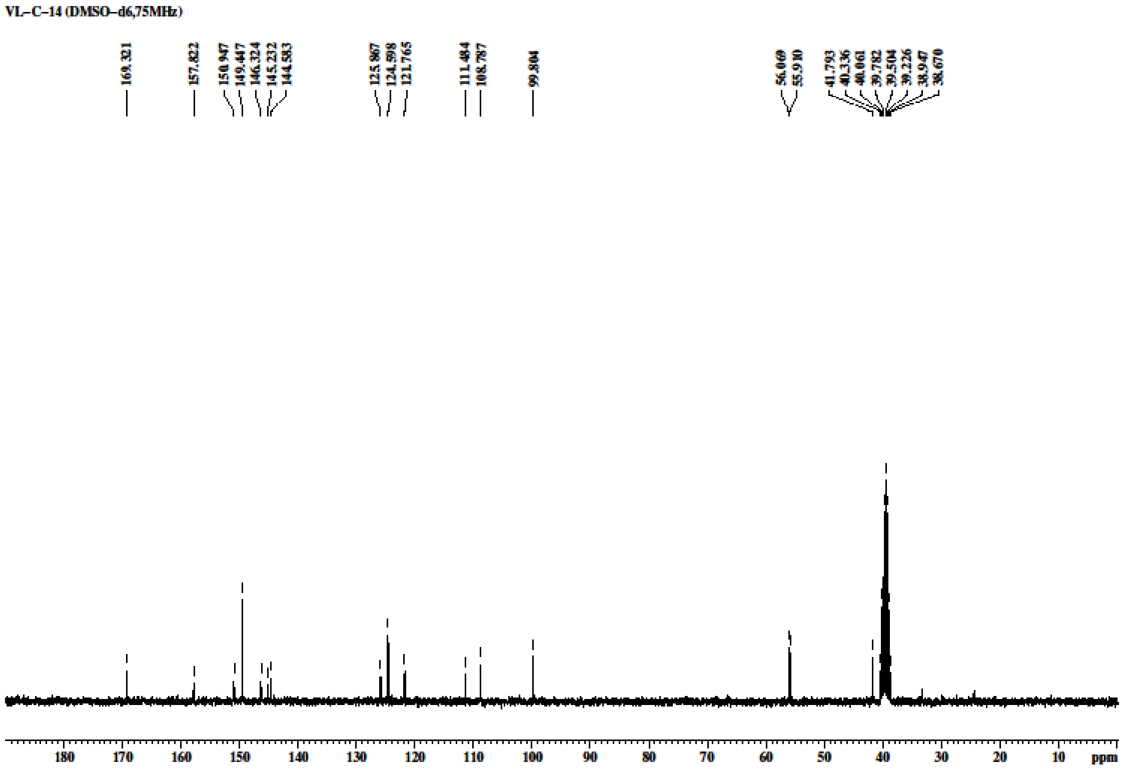

^1H and ^{13}C NMR (DMSO-d_6, 300 MHz) of compound **10a**

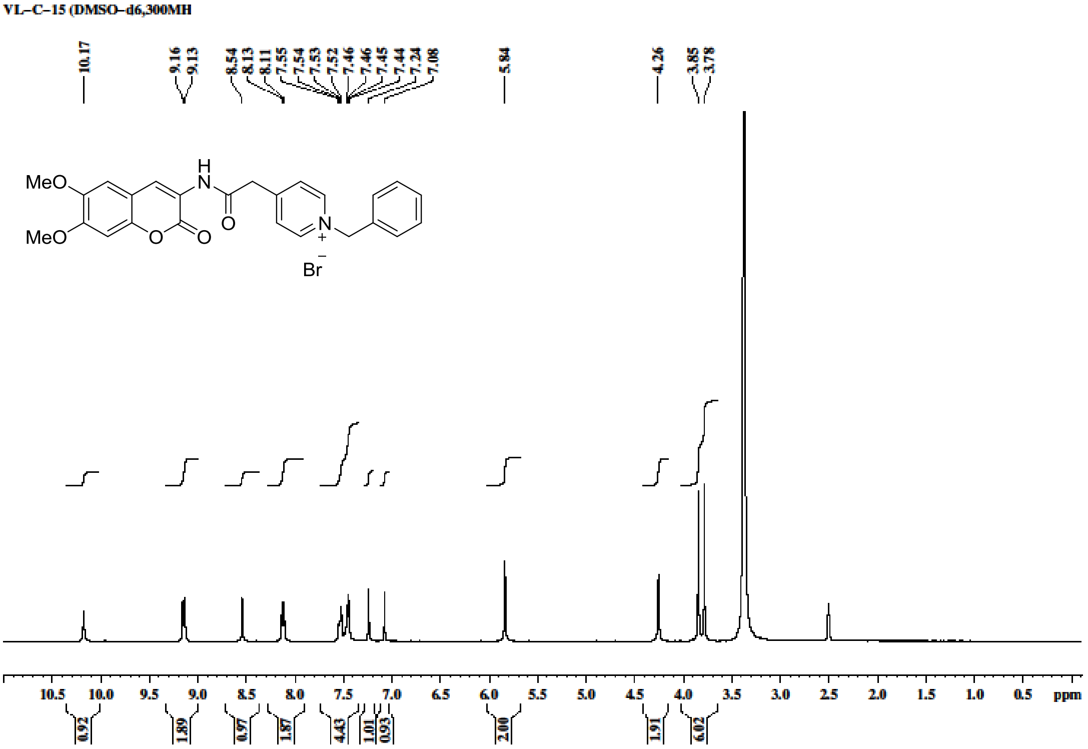

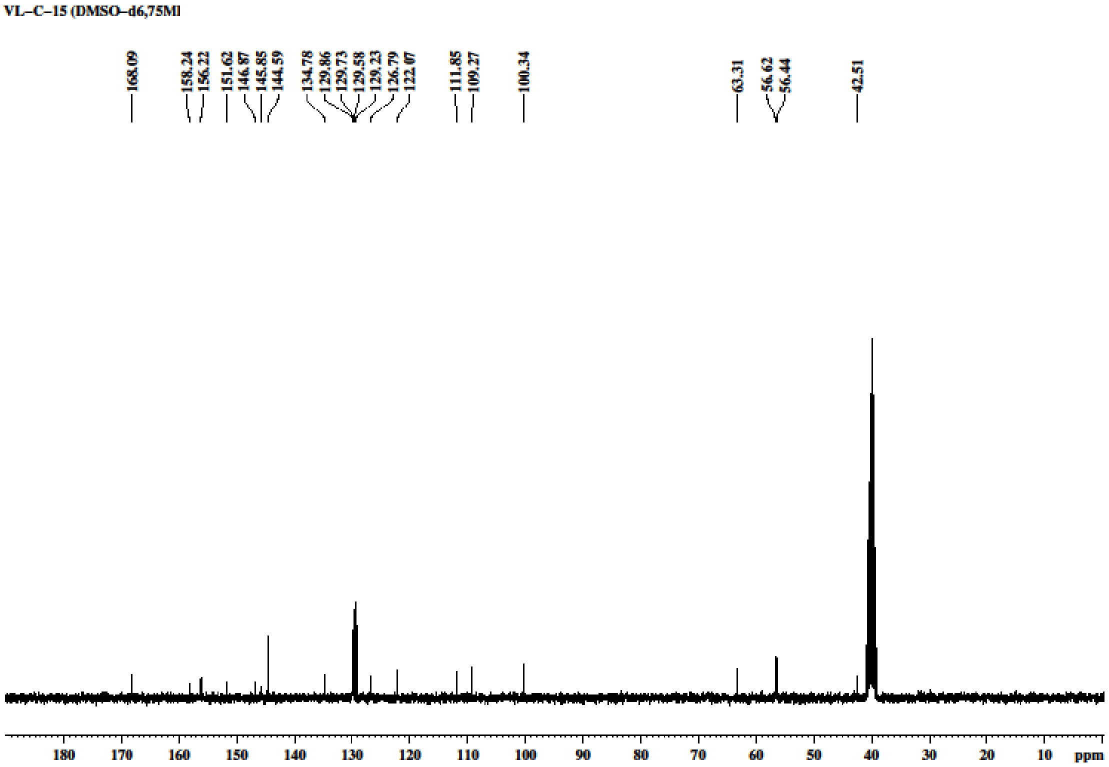

Supplement: File 1 — Experimental details, characterization data and copies of NMR spectra. [file Beilstein_J_Org_Chem-14-2545-s001.pdf]
